# T cell receptor usage and epitope specificity amongst CD8^+^ and CD4^+^ SARS-CoV-2-specific T cells

**DOI:** 10.3389/fimmu.2025.1510436

**Published:** 2025-02-28

**Authors:** Ulrik Fahnøe, Shan Feng, Alexander P. Underwood, Kivin Jacobsen, Amir Ameri, Thomas H. Blicher, Christina S. Sølund, Brad R. Rosenberg, Liselotte Brix, Nina Weis, Jens Bukh

**Affiliations:** ^1^ Copenhagen Hepatitis C Program (CO-HEP), Department of Infectious Diseases, Copenhagen University Hospital, Hvidovre, Denmark; ^2^ Copenhagen Hepatitis C Program (CO-HEP), Department of Immunology and Microbiology, Faculty of Health and Medical Sciences, University of Copenhagen, Copenhagen, Denmark; ^3^ Department of Infectious Diseases, Copenhagen University Hospital, Hvidovre, Denmark; ^4^ Immudex ApS, Virum, Denmark; ^5^ Department of Microbiology, Icahn School of Medicine at Mount Sinai, New York, NY, United States; ^6^ Department of Clinical Medicine, Faculty of Health and Medical Sciences, University of Copenhagen, Copenhagen, Denmark

**Keywords:** SARS-CoV-2, COVID-19, T cells, flow cytometry, single-cell RNA sequencing, transcriptomics, CD4^+^ -specific cells, CD8^+^ -specific cells

## Abstract

**Introduction:**

The coronavirus disease 2019 (COVID-19) pandemic, caused by severe acute respiratory syndrome coronavirus 2 (SARS-CoV-2), has highlighted the critical importance of understanding protective long-lasting immune responses. This study investigates the epitope specificity, T cell receptor (TCR) usage, and phenotypic changes in SARS-CoV-2-specfic CD8^+^ and CD4^+^ T cells over time in convalescent individuals with COVID-19.

**Methods:**

Peripheral blood mononuclear cells (PBMCs) were collected from 28 unvaccinated individuals with primary SARS-CoV-2 infection (6 identified as the D614G variant, clade 20C) and analyzed up to 12 months post-symptom onset. Antigen-specific CD8^+^ and CD4^+^ T cells were analyzed using flow cytometry and single-cell RNA sequencing (scRNAseq) using specific dextramer and antibody reagents. TCR clonotypes and activation markers were characterized to explore T cell dynamics.

**Results:**

SARS-CoV-2-specific CD8^+^ T cells exhibited waning frequencies long-term, transitioning from memory-like to a naïve-like state. scRNAseq revealed specificity against both spike and non-spike antigens with increased CD95 and CD127 expression over time, indicating that naïve-like T cells may represent stem cell memory T cells, which are multipotent and self-renewing, likely important for long-lived immunity. TCR clonal expansion was observed mainly in memory T cells, with overlapping TCR beta chain (TRB)-complementary determining region 3 (CDR3) sequences between participants, suggesting shared public TCR epitope-specific repertoires against SARS-CoV-2. Further, unique spike-specific CD4^+^ T cells with high CD95 and CD127 expression were identified, which may play a crucial role in long-term protection.

**Discussion:**

This study highlights epitope-specificity heterogeneity, with some immunodominant responses, and suggests a potential role for long-lived SARS-CoV-2-specific T cell immunity. Shared TCR repertoires offers insights into cross-reactive and protective T cell clones, providing valuable information for optimizing vaccine strategies against emerging SARS-CoV-2 variants. The findings underscore the critical role of cellular immunity in long-term protection against SARS-CoV-2 and emphasizes the importance of understanding T cell dynamics.

## 1 Introduction

Since late 2019, the pandemic caused by severe acute respiratory syndrome coronavirus-2 (SARS-CoV-2) and its associated disease, coronavirus disease 2019 (COVID-19), has caused millions of deaths worldwide (1). After the worldwide distribution of various vaccines against COVID-19 in 2021, the burden of disease and its associated mortality has been greatly reduced (1). However, unlike other classical viral vaccines, which can establish long-lasting protective immunity, such as those that protect against polio, measles, mumps and rubella, vaccination against COVID-19 appears to generate shorter-lived protection (2) like influenza (flu) vaccines (3). The shorter-lived immunity seen in both COVID-19 and flu vaccines is not only due to waning immunity, but also due to the emergence of antigenically distinct variants and strains capable of evading prior immunity (4, 5). Because of this, booster vaccine regimens containing updated formulas that target the dominant circulating variants have been implemented to improve protection efficacy against COVID-19 (6, 7). To induce long-lasting protection against COVID-19, it is important to understand the long-lived immune responses generated by SARS-CoV-2 infection, which, in turn, may help better guide vaccine designs.

For most vaccines, it is thought that their successful protection against disease is attributed to the generation of neutralizing antibody (nAb) responses. Similarly, nAb responses have been shown to be a strong correlate of protection against developing severe COVID-19 (8–10). However, unlike most other vaccines, vaccination against COVID-19 has been shown to also induce cellular immune responses, which have also been linked as a correlate of protection (11). Compared to nAb responses, cellular responses against SARS-CoV-2 are more durable in peripheral blood (12, 13), but unlike nAb responses, which have diminished efficacy against emerging SARS-CoV-2 variants (14, 15), epitopes specific to the cellular immune response have been found to be highly conserved amongst the different SARS-CoV-2 variants (12, 16, 17). Thus, it is highly likely that cellular immunity against SARS-CoV-2 will be essential for long-term protection.

Although the cellular immune response is very broad, it can generally be divided into two major arms: the helper T cell response (CD4^+^ T cells) and the killer T cell response (CD8^+^ T cells). Unlike antibody epitopes, which may be conformational and cover long or discontinuous stretches of amino acids, T cell receptors (TCRs) recognize epitopes spanning 9-15 amino acids in length presented on major histocompatibility (MHC) molecules. The peptides presented on MHC molecules are restricted by the host genotype (also called human leukocyte antigen [HLA] type), and therefore the specific set of peptides presented differ greatly between individuals. However, large research efforts on viruses that are highly exposed to the human population, such as cytomegalovirus (CMV), Epstein-Barr virus (EBV) and flu, has revealed immunodominant epitopes presented by the different HLA types for these viruses (18–20). In the context of SARS-CoV-2, several studies have deconvoluted epitope specificities within certain HLA types (21, 22). For vaccinated, infection-naïve individuals, these epitopes are primarily restricted to the virus’ spike (S) protein since this is the principal viral component in most COVID-19 vaccines. For unvaccinated, infection-experienced individuals, SARS-CoV-2-specific T cell epitopes appear to span the entire viral proteome with some evidence of a single epitope giving rise to an immunodominant response (23–26).

**Figure 1:**
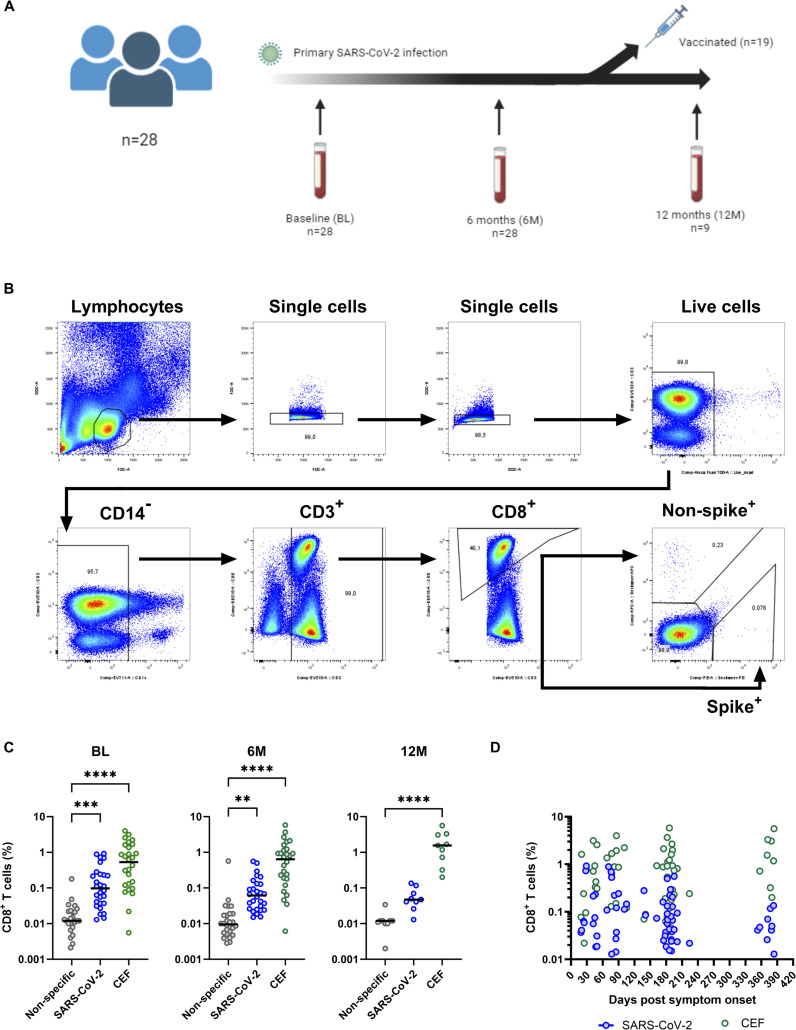
SARS-CoV-2-specific CD8^+^ T cell responses after primary SARS-CoV-2 infection. **(A)** Schematic showing the timeline of the 28 individuals selected for this study. The blood tubes indicate the collection time point and the syringe indicates those that received a COVID-19 vaccination and therefore were excluded from analysis at the 12M time point. **(B)** The gating strategy used for identification of antigen-specific CD8^+^ T cells. PBMCs were gated on lymphocytes, single cells, live cells, CD14^-^, CD3^+^, CD8^+^ and then either APC or PE positive as indicated. **(C)** Comparison of non-specific (grey circles), SARS-CoV-2-specific (blue circles) and CMV, EBV and flu (CEF)-specific (green circles) CD8^+^ T cell frequencies at the BL (left), 6M (middle) and 12M (right) time points. **(D)** Frequencies of SARS-CoV-2-specific (blue circles) and CEF-specific (green circles) CD8^+^ T cells plotted longitudinally according to the days post-symptom onset (X axis). Comparisons of CD8^+^ T cell frequencies was done using the Friedman test and corrected using Dunn’s test for multiple comparisons. Significant differences are shown as **p < 0.01, ***p < 0.001 and ****p < 0.0001.

Several studies have demonstrated the durability of SARS-CoV-2-specific CD8^+^ T cell responses following SARS-CoV-2 infection (27–31) and COVID-19 vaccination (30–34). However, details are lacking concerning the long-term protective potential of SARS-CoV-2-specific T cells and on TCR usage amongst SARS-CoV-2-specific T cells, which can be extremely valuable for identifying publicly shared, cross-reactive, long-lasting, protective T cell clones. Furthermore, as all of this is related to CD8^+^ T-cells, data for SARS-CoV-2-specific CD4^+^ T-cells are very limited. To address these questions, characterization of antigen-specific CD8^+^ T cells in 28 unvaccinated, non-hospitalized, convalescent individuals after a primary SARS-CoV-2 infection with follow-up at six (6M) and twelve months (12M)-post symptom onset was performed (**Figure 1A**). Following this, single-cell RNA sequencing (scRNAseq) was performed on antigen-specific CD4^+^ and CD8^+^ T cells from four selected individuals for in-depth analysis through deconvolution of epitope specificities, transcriptomic data, and TCR usage.

## 2 Materials and methods

### 2.1 Study cohort

Participants for this study were selected from the Clinical, Virological and Immunological COVID-19 (CVIC) study, Department of Infectious Diseases, Copenhagen University Hospital, Hvidovre, Denmark, which is a prospective cohort of individuals either infected by SARS-CoV-2 and/or vaccinated against COVID-19. Details of this cohort have been previously described (10, 34–36). All individuals included in this study were selected based on confirmation of a primary SARS-CoV-2 infection in the absence of COVID-19 vaccination. Furthermore, any infections that resulted in hospital admittance were excluded from this study. Confirmation of SARS-CoV-2 infection was done via routine diagnostic polymerase chain reaction (PCR) or via screening of anti-S or anti-nucleocapsid (N) antibodies (outlined below). Longitudinal follow up included blood collection at enrolment (baseline [BL]), 6M- and 12M-post symptom onset (**Figure 1A**; **Supplementary Table S1**). Any time points after the individual had received a COVID-19 vaccination were excluded. Blood was collected in ethylenediaminetetraacetic acid (EDTA) collection tubes and processed using Ficoll density grade separation to isolate and cryopreserve plasma and peripheral blood mononuclear cells (PBMCs) at -80°C and -150°C, respectively. SARS-CoV-2 sequences, where possible, were retrieved from NGS data stored at the Department of Clinical Microbiology, Copenhagen University Hospital, Hvidovre, Denmark.

### 2.2 Study approval

All individuals included in this study were 18 years or older and able to read and speak adequate Danish to provide written informed consent. This study was approved by the Regional Ethical Committee (H-20025872) and Data Protection Agency (P-2020-357) and was conducted in compliance with the Declaration of Helsinki guidelines. Study data was collected and managed using research electronic data capture (REDCap) tools (37).

### 2.3 Enzyme-linked immunosorbent assays

All enzyme-linked immunosorbent assays (ELISAs) were done previously (10, 35). In brief, assessment of anti-SARS-CoV-2 RBD and anti-SARS-CoV-2 N antibodies was done using a WANTAI SARS-CoV-2 antibody ELISA kit and EuroImmun ELISA kit (PerkinElmer), respectively.

Quantitative assessment of plasma-derived SARS-CoV-2 S-specific IgA and IgG was done previously (35). In brief, SARS-CoV-2 S protein was collected from HEK293T cell lysates and added to NUNC Maxisorp plates coated with *Galanthus nivalis* (GNA) lectin. Blocking was done with non-fat dairy milk protein. Serially diluted plasma was added, and detection of anti-spike IgG and IgA was done using anti-human IgG and anti-human IgA secondary antibodies conjugated with horseradish peroxidase (HRP). Subsequently, 3,3’,5,5’-tetramethylbenzidine (TMB) was added and the reaction was stopped after 15 minutes with 1M hydrochloric acid (HCl). The absorbance was then detected at 450nm using an ELx808 Ultra Microplate Reader (BioTek Instruments).

### 2.4 Neutralization assay

The subject’s used in this study have previously been analyzed for neutralizing antibody titers in the corresponding serum sample to a SARS-CoV-2 isolate DK-AHH1 as described (35, 36, 38). In brief, heat inactivated, 2-fold serially diluted plasma was incubated with SARS-CoV-2 isolate DK-AHH1 (MOI of 0.01 for 10^4^ cells) at a 1:1 ratio for 1 hour at room temperature. Following this, the plasma/virus mix was added in quadruplicate to 10^4^ Vero E6 cells seeded the day before and incubated at 37°C and 5% CO_2_ for 48 hours. The cells were then washed, fixed, and stained as previously described (35). Spots representing infected cells were counted using an Immunospot series 5 UV analyzer (Cellular Technologies) and the percentage neutralization was calculated by comparing the spot count of the plasma dilution to the pooled healthy plasma control as previously described (35).

### 2.5 Human leukocyte antigen typing

HLA typing of subjects was done using a protocol previously developed by others (39). In brief, subject DNA was obtained by lysing one vial of PBMCs using a DNeasy Blood & Tissue kit (Qiagen) according to the manufacturer’s instructions. This DNA was used as a template for PCR amplification of the HLA A, HLA B and HLA DRB1 alleles using the primers and PCR cycling conditions previously described (39). Following PCR, the products were checked via gel agarose electrophoresis and purified using AMPure XP beads (Qiagen) according to the manufacturer’s instructions. Purified products were then pooled, and library preparation was conducted using a NEBNext Ultra II DNA Library Preparation kit (New England Biolabs). Next generation sequencing (NGS) was performed using the MiSeq platform (Illumina). The data was analyzed using Hisat-genotype analysis pipeline as described (40). To validate these results, HLA typing was also done using an AlloSeq Tx kit (CareDx) according to the manufacturer’s instructions.

### 2.6 Flow cytometry for the identification of antigen-specific CD8^+^ T cells

Frozen PBMCs were thawed at room temperature, transferred to phosphate buffered saline (PBS) containing 5% fetal calf serum (FCS) and centrifuged at 300 x *g* for 10 min. The cell pellet was resuspended in PBS containing 5% FCS and stained with fixable viability stain (FVS) 700 (Alexa Fluor^®^ 700, BD Biosciences) for 20 min in the dark. The PBMCs were washed twice and resuspended in Brilliant Stain Buffer (BD Biosciences). The cells were then split equally into two separate wells and stained with Dextramer^®^ reagents according to the manufacturer’s instructions. The first well of cells was stained with SARS-CoV-2-specific Dextramer^®^ reagents and the second well of cells was stained with non-specific Dextramer^®^ reagents (negative control, APC labelled) and CMV, EBV and flu (CEF)-specific Dextramer^®^ reagents (PE labelled; see **Supplementary Table S2** for a full list of Dextramers). A summary of the Dextramer^®^ reagents used for each subject can be found in **Supplementary Table S3**. Following Dextramer^®^ staining, the cells were stained in the dark for 20 min with anti-CD3 (V500, BD Biosciences), anti-CD8 (BB515, BD Biosciences), anti-CD14 (BV711, BD Biosciences), anti-CD45RA (APC-H7, BD Biosciences), anti-HLA-DR (PerCp-Cy5.5, BD Biosciences), anti-CD38 (BUV395, BD Biosciences), anti-CD27 (BV786, BD Biosciences), anti-CD127 (PE-Cy7, BD Biosciences), anti-PD-1 (BV421, BD Biosciences), and anti-CD197 (CCR7) (BV650, BD Biosciences). The cells were then washed four times and resuspended in 200 μl PBS containing 5% FCS. The samples were then analyzed on an BD LSRFortessa X20 Analyzer (BD Biosciences). Flow cytometry data was analyzed using FlowJo software (version 10.8.1). Samples were gated as shown in **Figure 1B**.

### 2.7 Fluorescence-activated cell sorting of SARS-CoV-2-specific T cells

Frozen PBMCs were thawed at room temperature, transferred to PBS containing 5% FCS and centrifuged at 300 x *g* for 10 min. The cells were resuspended in PBS containing 5% FCS and stained with FVS700 for 20 min in the dark. Subsequently, cells were resuspended in PBS containing 2% FCS and 0.1 g/L Herring sperm DNA (Promega #1811, 10 mg/ml), and incubated in the dark for 30 min with dCODE Dextramer^®^ reagents, including MHC class I dCODE Dextramer^®^ reagents of MHC-peptide combinations as outlined in **Supplementary Table S4**, and MHC class II dCODE Dextramer^®^ reagents as listed in **Supplementary Table S5**. Following this, the cells were stained in the dark for 20 min with anti-CD3 (APC, BD Biosciences) and TotalSeq™ antibodies (Biolegend), including anti-CD3, anti-CD4, anti-CD8, anti-CD45RA, anti-HLA-DR, anti-PD-1, anti-CCR7, anti-CD38, anti-CD27, anti-CD95, anti-CD14, anti-CD127, and a mouse IgG1 κ isotype control. The cells were then washed twice with PBS containing 2% FCS, resuspended in PBS containing 50% FCS and strained through the meshed snap cap into a flow cytometry tube. CD3^+^ Dextramer^+^ cells were sorted using a BD FACSARIA II cell sorter (BD Biosciences). Sorted antigen-specific cells were then used immediately for scRNAseq using the Chromium platform (10X genomics).

### 2.8 Chromium single-cell RNA sequencing of antigen-specific T cells

Sorted cells were manually counted and set to maximum input of 10,000 cells before loading into the Chromium sequencer using Chromium Next GEM Single Cell 5’ Reagents Kits v2 (Dual Index, 10x Genomics). Subsequently, cDNA synthesis and double-stranded synthesis were performed as suggested by the manufacturer. Libraries for scRNAseq, scTCRseq and scCITEseq were constructed, measured, and normalized by Qubit (BMG Labtech) and a Bioanalyzer (Agilent). Sequencing was performed on Novaseq (Illumina), multiplexing the 3 library types of the 12 samples in differentially equimolar amounts.

### 2.9 Single-cell RNA sequencing data analysis

Cell Ranger (10X Genomics version 5.0) was used to demultiplex the sequencing data and subsequently analyzing the scRNAseq, scTCRseq and scCITEseq datasets for each sample using the multi algorithm in the software pipeline. The scCITEseq data was pre-processed and normalized by dsb software (version 1.0.3) (41). The Seurat tool (version 5) was used in R Studio (version 2023.03.0 + 386) to normalize, transform, and cluster the scRNAseq and the normalized scCITEseq data, and for subsequent visualization. Data was transformed applying SCTransform v2 and subsequent integration of all samples was performed after removing all TCR genes from the integration features. UMAP projection was performed on either scRNAseq or scCITEseq individually as indicated in the figure legends using Seurat WNN functionality. ScTCRseq analysis was performed by scRepertoire (version 2.0.0) (42) and integrated into the Seurat object to evaluate individual cells TCR clonotype expansion. Data was further investigated for T cells specific to the TTDPSFLGRY (ORF1ab) within and between participants NH09 and NH52 by analysis of TRB-CDR3 overlap, TRB-CDR3 amino acid composition and 5-mer usage and T cell receptor beta variable (TRBV) gene usage. Visualization of the TCR analysis was also performed with scRepertoire.

### 2.10 Statistics

Statistical analyses were performed with GraphPad Prism (version 9.5.1). Categorical variables were summarized with count and proportion (n, %) and continuous variables with median value and interquartile range (IQR) or mean value with standard deviation (SD). Normal distribution was analyzed using QQ-plots and assessed using the Shapiro-Wilk test and the Kolmogorov-Smirnov test. Paired data that was not found to be normally distributed was compared using two-tailed Wilcoxon t tests and Friedman tests. Non-paired data that was not found to be normally distributed was analyzed using Mann-Whitney or Kruskal-Wallis tests. Corrections for multiple comparisons was done using Dunn’s test. Correlations between two continuous variables were assessed using the Pearson correlation coefficients. Statistical significance was determined as a *p* value of less than 0.05.

## 3 Results

### 3.1 Study participants

Of the 102 participants that acquired a non-hospitalized primary SARS-CoV-2 infection in the CVIC cohort (10, 34, 35), 28 were selected for this study. This selection was based on good participant follow-up and individuals having desirable HLA alleles, which included A*01:01, A*02:01, A*03:01, A*11:01, A*24:02, B*07:02, B*08:01, and B*35:01. Blood had been collected from all 28 selected participants at baseline (BL, 0.5-5 months (M) post-symptom onset), 6M post-symptom onset and 12M post-symptom onset (**Figure 1A**). At the BL and 6M time points, none of the participants had received a COVID-19 vaccination. However, at the 12M time point, 19 had received a COVID-19 vaccination and were excluded from analysis at this time point (**Figure 1A**). The median age at enrolment was 41 years (IQR=28-51 years) and 20 (71%) were female. A summary of the 28 selected participants can be found in **Supplementary Table S1**.

### 3.2 Longitudinal identification of antigen-specific CD8^+^ T cells after primary SARS-CoV-2 infection

To identify antigen-specific CD8^+^ T cells, Dextramer reagents loaded with MHC class I epitopes corresponding to the SARS-CoV-2 S protein, other SARS-CoV-2 proteins (termed “non-S”), immunodominant epitopes to CEF and to non-specific epitopes (termed “negative control”) were selected for HLA A*01:01, A*02:01, A*03:01, A*11:01, A*24:02, B*07:02, B*08:01 and B*35:01.

Identification of antigen-specific CD8^+^ T cells was done by preparing PBMCs into two pools and gated as shown in **Figure 1B**. The first pool of PBMCs was made to identify SARS-CoV-2-specific CD8^+^ T cells using S protein-specific Dextramer reagents (phycoerythrin [PE] labelled) and non-S protein-specific Dextramer reagents (allophycocyanin [APC] labelled). The second pool of PBMCs was made to identify CEF-specific (PE labelled) and non-specific (APC labelled, negative control) CD8^+^ T cells. Initially, identified frequencies of S protein-specific and non-S protein-specific CD8^+^ T cells were compared to see if S protein-specific epitopes were more immunodominant than non-S protein-specific epitopes (**Supplementary Figure S1**). However, no differences were found between the frequencies of both S protein-specific and non-S protein-specific CD8^+^ T cells and thus these were combined for further analyses (termed “SARS-CoV-2-specific CD8^+^ T cells”).

To understand if the identified frequencies of SARS-CoV-2-specific CD8^+^ T cells were above non-specific binding levels, these frequencies were compared to the identified frequencies against the non-specific Dextramer and CEF-specific CD8^+^ T cell frequencies at each time point (**Figure 1C**). When compared to the non-specific Dextramer CD8^+^ T cell frequencies, both the SARS-CoV-2-specific and CEF-specific CD8^+^ T cell frequencies were significantly higher at the BL (*p*<0.001, Kruskal-Wallis test) and 6M time points (*p*<0.01, Kruskal-Wallis test). However, at the 12M time point, while the SARS-CoV-2-specific CD8^+^ T cell frequencies were close to significance (*p*=0.0534, Kruskal-Wallis test), only the CEF-specific CD8^+^ T cell frequencies were significantly higher than the non-specific Dextramer CD8^+^ T cell frequencies (p<0.0001, Kruskal-Wallis test). When the SARS-CoV-2-specific and CEF-specific CD8^+^ T cell frequencies were compared, significantly higher CEF-specific CD8^+^ T cell frequencies were detected at the 6M time point (*p*=0.0200, Kruskal-Wallis test); BL and 12M were not found to be significantly different.

Next, the frequencies of the SARS-CoV-2-specific and CEF-specific CD8^+^ T cells were analyzed over time (**Figure 1D**). For SARS-CoV-2-specific CD8^+^ T cell frequencies, higher levels were identified closer to the infection date. At the 6M time point, these frequencies were observed to be lower, with even lower frequencies observed at the 12M time point indicating a waning of these responses over time. By comparison, CEF-specific CD8^+^ T cell frequencies were found to be maintained across all three time points. When the frequencies were analyzed by time points, frequencies of SARS-CoV-2-specific CD8^+^ T cells were found to be significantly higher at BL compared to the 6M and 12M time points (*p*<0.05, **Supplementary Figure S2**).

Given that neutralizing data, as well as IgG and IgA data, had been previously collected for these individuals (35), these data were compared to the SARS-CoV-2-specific CD8^+^ T cell frequencies at every time point. It is important to note that IgG and IgA data had not been collected at the 12M time point for these individuals and, thus, only the BL and 6M time points were compared to their respective SARS-CoV-2-specific CD8^+^ T cell frequencies. No correlations were found between SARS-CoV-2-specific CD8^+^ T cell frequencies and neutralizing titers (r=0.099, *p*=0.921), anti-spike protein plasma IgG levels (r=0.056, *p*=0.679) or anti-spike protein plasma IgA levels (r=0.233, *p*=0.084) (**Supplementary Figure S3**).

### 3.3 Measuring activation markers over time in identified antigen-specific CD8^+^ T cells

**Figure 2:**
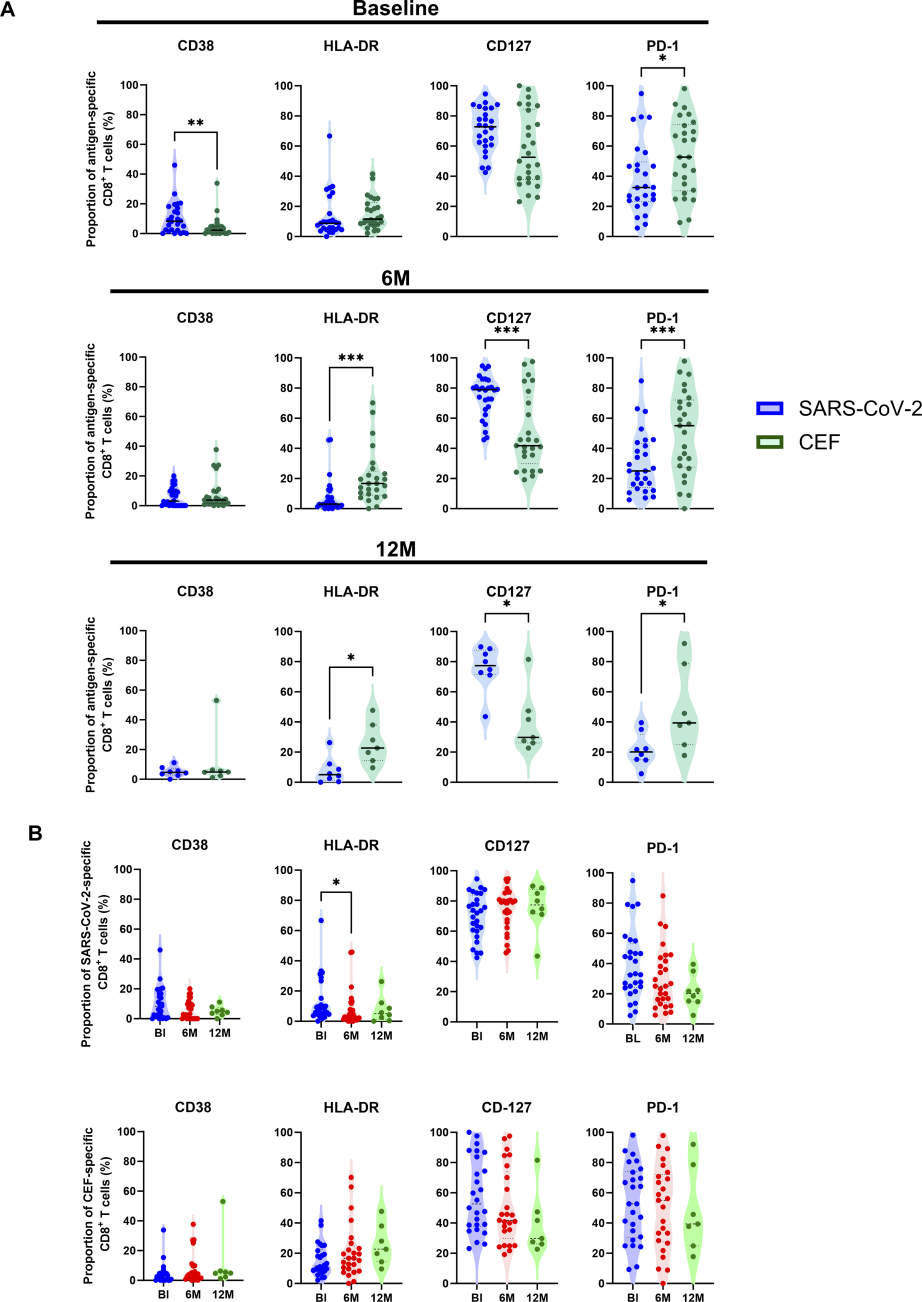
Characterization of activation marker expression in antigen-specific CD8^+^ T cells. **(A)** Comparison of the proportion (%) of SARS-CoV-2-specific (blue circles) to CEF-specific (green circles) CD8^+^ T cells expressing either CD38, HLA-DR, CD127 and PD-1 at BL (top), 6M (middle) and 12M (bottom). **(B)** Comparison of the proportion (%) of SARS-CoV-2-specific (top) and CEF-specific (bottom) CD8^+^ T cells between the BL (blue), 6M (red) and 12M (green) time points. Comparisons of the proportions of expression between SARS-CoV-2-specific and CEF-specific CD8^+^ T cells were done using Wilcoxon t tests. Comparisons of the proportions of expression over the BL, 6M and 12M time points was done using Kruskal-Wallis tests and corrected using Dunn’s test for multiple comparisons. Significant differences are shown as *p < 0.05, **p<0.01 and ***p < 0.001.

To characterize the activation status in identified antigen-specific CD8^+^ T cells, expression of CD38, HLA-DR, CD127 and PD-1 was analyzed over time (**Figure 2**). The proportion of antigen-specific CD8^+^ T cells positive for these markers was firstly compared between SARS-CoV-2-specific and CEF-specific CD8^+^ T cell populations (**Figure 2A**). At the BL time point, significantly higher proportions of CD38^+^ T cells were found in the SARS-CoV-2-specific CD8^+^ T cell population when compared to the CEF-specific CD8^+^ T cell population (*p*=0.0027, Wilcoxon T test). In contrast, significantly higher proportions of PD-1^+^ T cells were found in the CEF-specific CD8^+^ T cell population when compared to the SARS-CoV-2-specific CD8^+^ T cell population at this time point (*p*=0.0275, Wilcoxon T test). At both the 6M and 12M time points, significantly higher proportions of HLA-DR^+^ and PD-1^+^ T cells were found in the CEF-specific CD8^+^ T cell populations. In contrast, significantly higher proportions of CD127^+^ T cells were found in the SARS-CoV-2-specific CD8^+^ T cell populations at these time points. When the SARS-CoV-2-specific CD8^+^ T cell populations were compared over time (**Figure 2B**), the proportion of HLA-DR^+^ T cells was found to significantly decrease from the BL time point to the 6M time point (*p*=0.0494, Kruskal-Wallis test). While there was an observable decrease in CD38^+^ T cells over time, this didn’t reach significance (*p*>0.05, Kruskal-Wallis test). Of note, the proportion of CD127^+^ T cells was not observed to change over time. In the CEF-specific CD8^+^ T cell populations, no significant differences were observed for any activation marker over time.

### 3.4 Characterization of antigen-specific CD8^+^ T cell subsets over time

**Figure 3:**
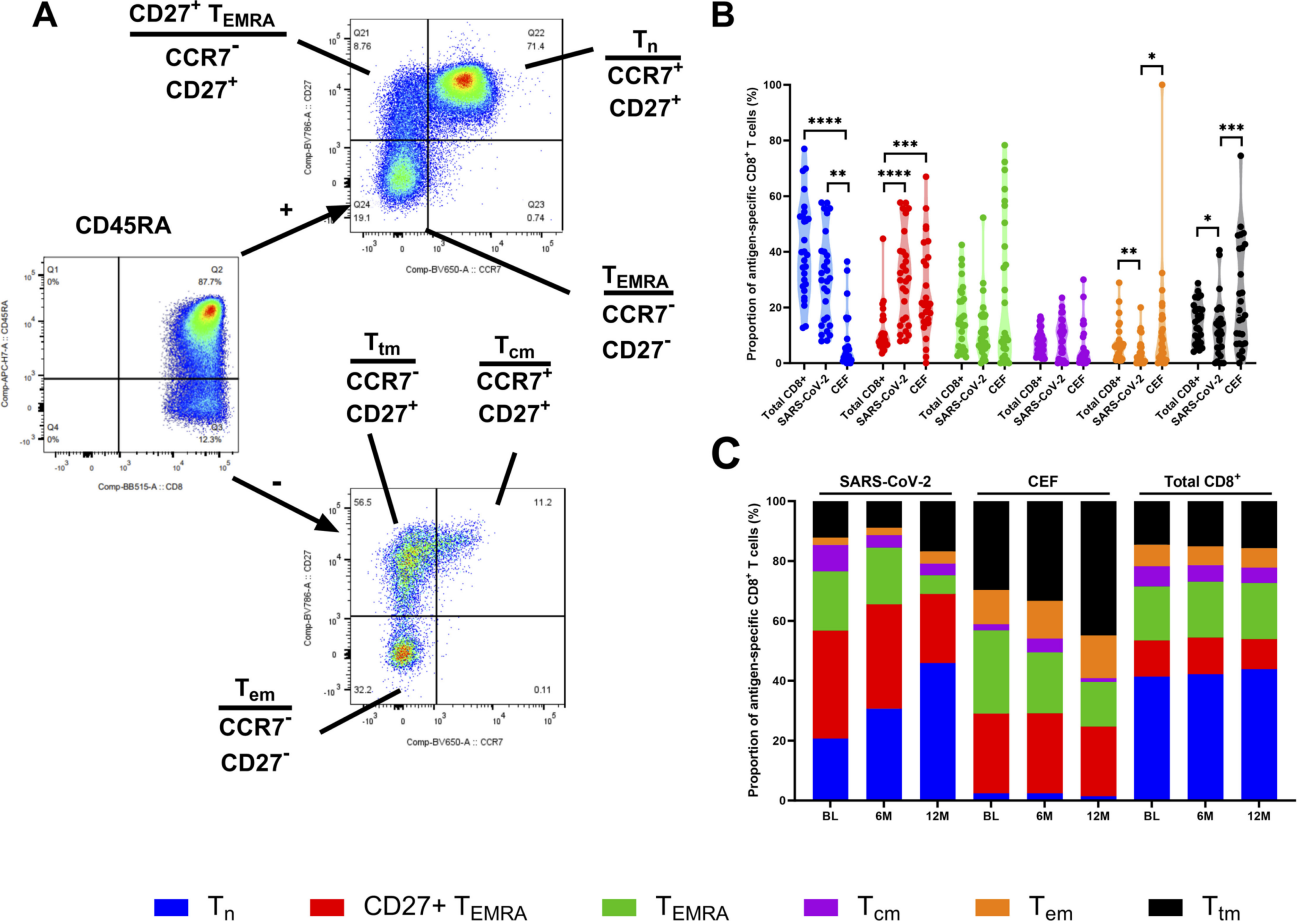
Phenotyping of antigen-specific CD8^+^ T cell subsets. **(A)** Representative gating strategy to define different memory CD8^+^ T cell subsets. Cells were firstly gated on CD45RA and then on CCR7 and CD27. Cell subsets were defined as T_n_ (CD45RA^+^, CCR7^+^ and CD27^+^), CD27^+^ T_EMRA_ (CD45RA^+^, CCR7^-^ and CD27^+^), T_EMRA_ (CD45RA^+^, CCR7^-^ and CD27^-^), T_cm_ (CD45RA^-^, CCR7^+^ and CD27^+^), T_tm_ (CD45RA^-^, CCR7^-^ and CD27^+^) and T_em_ (CD45RA^-^, CCR7^-^ and CD27^-^). **(B)** The proportion (%) of T_n_ (blue), CD27^+^ T_EMRA_ (red), T_EMRA_ (green), T_cm_ (purple), T_em_ (orange), T_tm_ (black) in the total CD8^+^ T cell population, the SARS-CoV-2-specific CD8^+^ T cells and CEF-specific CD8^+^ T cells at the BL time point. **(C)** Stacked histogram showing the total proportions (%) of T_n_ (blue), CD27^+^ T_EMRA_ (red), T_EMRA_ (green), T_cm_ (purple), T_em_ (orange), T_tm_ (black) in the SARS-CoV-2-specific CD8^+^ T cells, CEF-specific CD8^+^ T cells and total CD8^+^ T cell population at the BL, 6M and 12M time points. Comparisons of the proportions of T cell subsets between the total CD8^+^ T cell population, the SARS-CoV-2-specific CD8^+^ T cells and CEF-specific CD8^+^ T cells was done using Kruskal-Wallis tests and corrected using Dunn’s test for multiple comparisons. Significant differences are shown as *p < 0.05, **p <0.01, ***p < 0.05 and ****p < 0.001 for B.

To investigate the distribution and differentiation of T cell subsets among antigen-specific CD8^+^ T cells, expression of CD45RA, CCR7 and CD27 was measured. T cell subsets were characterized as naïve cells (T_n_; CD45RA^+^, CCR7^+^, CD27^+^), terminally differentiated effector memory cells (T_EMRA_; CD45RA^+^, CCR7^-^, CD27^-^), CD27^+^ T_EMRA_ (CD45RA^+^, CCR7^-^, CD27^+^), central memory cells (T_cm_; CD45RA^-^, CCR7^+^, CD27^+^), transitional memory cells (T_tm_; CD45RA^-^, CCR7^-^, CD27^+^) and effector memory cells (T_em_; CD45RA^-^, CCR7^-^, CD27^-^), as indicated in **Figure 3A**. The proportion of these T cell subsets was then compared between the SARS-CoV-2-specific, CEF-specific and total CD8^+^ T cell populations (**Figure 3B**). At the BL timepoint, when the SARS-CoV-2-specific and CEF-specific CD8^+^ T cell populations were compared, a significantly higher proportion of T_n_ cells was found in the SARS-CoV-2-specific CD8^+^ T cell population (*p*=0.0009, Friedman test), and significantly higher proportions of T_tm_ and T_em_ cells were found in the CEF-specific CD8^+^ T cell population (*p*=0.0002 and *p*=0.0292, respectively; Friedman tests). When both the SARS-CoV-2-specific and CEF-specific CD8^+^ T cell populations were compared to the total CD8^+^ T cell population, significantly higher proportions of CD27^+^ T_EMRA_ cells were found (*p*<0.0001, Friedman test). Significantly higher proportions of T_n_ cells were also found in the total CD8^+^ T cell population when compared to the CEF-specific CD8^+^ T cell population (*p*<0.0001, Friedman test). Significantly higher proportions of T_em_ cells were found in the total CD8^+^ T cell population when compared to the SARS-CoV-2-specific CD8^+^ T cells (*p*=0.0052, Friedman test). Inversely, significantly higher proportions of T_tm_ cells were found in the SARS-CoV-2-specific CD8^+^ T cells when compared to the total CD8^+^ T cell population (*p*=0.0292, Friedman test). When the T cell subsets were analyzed over time (**Figure 3C**), reduced proportions of T_EMRA_ cells and increased proportions of T_n_ cells were found in the SARS-CoV-2-specific CD8^+^ T cell populations. In the CEF-specific CD8^+^ T cell populations, reduced proportions of T_EMRA_ cells and increased proportions of T_tm_ cells were found over time. In the total CD8^+^ populations, no differences in the proportions of the T cell subsets were observed over time.

### 3.5 Deconvolution of epitope specificities using single-cell RNA sequencing

**Figure 4:**
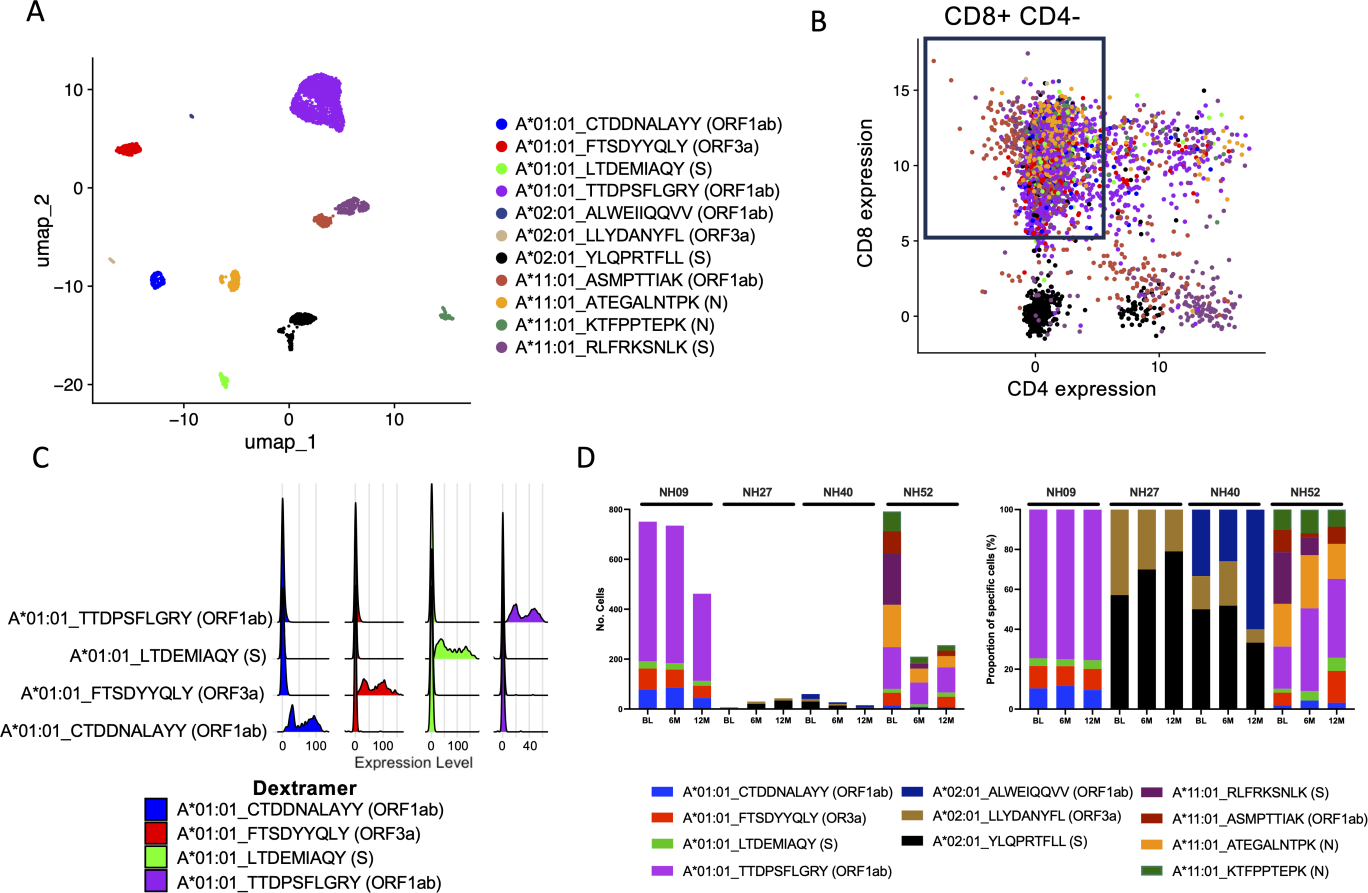
Deconvolution of SARS-CoV-2-specific CD8^+^ T cell epitopes through scRNAseq. **(A)** UMAP of identified SARS-CoV-2-specific CD8^+^ T cells for all four subjects at all time points (BL, 6M and 12M) using CITE-seq antibodies and Dextramer specificity with each single cell represented as a single dot. Each color represents a single epitope as indicated in the figure. **(B)** Scatterplot showing the CITE-seq expression of CD8 and CD4 among sorted SARS-CoV-2-specific CD3^+^ cells. Each color represents a single epitope matched to the figure legend in **(A)**. The box shows the gating of cells expressing CD8^+^ exclusively (low expression of CD4^+^) which were further examined. **(C)** Ridgeplot showing the specificity of the identified Dextramer-bound A*01:01-specific CD8^+^ T cells for participants NH09 and NH52. Each color represents a single epitope as indicated in the figure. The X axis represents the relative normalized binding of each Dextramer within each cluster identified in **(A)**, thus showing the specificity of the Dextramer signal within each cluster. **(D)** The total number of identified SARS-CoV-2-specific CD8^+^ T cells identified compared to total CD8^+^/CD4^-^ for each participant at each time point (left) and the proportion (%) of SARS-CoV-2-specific deconvoluted epitope specificities (right). Each color represents a single epitope as indicated in the figure.

To further investigate antigen-specific CD8^+^ T cells, the three time points from 4 participants (NH09, NH27, NH40 and NH52) matching either A*01:01, A*02:01, A*03:01 or A*11:01 epitopes (**Supplementary Table S4**) from the 9 participants that had a 12M time point included were selected for scRNAseq using the Chromium platform (10X genomics). Following normalization and visualization by UMAP, clustering analysis of CITE-Seq identified epitope specific cells (**Supplementary Figures S4A, B**), and the epitope specificity of each Dextramer-bound single cell was successfully deconvoluted for all four participants (**Figure 4A**; **Supplementary Figures S4C, D**). Given that cells were initially sorted on CD3 alone, CITE-Seq analysis of CD4 and CD8 expression among Dextramer-bound single cells showed that a large proportion of these cells either did not express CD8 or were found to express both CD4 and CD8 (**Figure 4B**). Therefore, to remove noise, only cells that exclusively expressed CD8 were investigated further (**Figure 4B**). Analysis of the antigen-specific CD8^+^ T cell populations showed that they were highly specific to the respective epitopes (**Figure 4C**; **Supplementary Figures S4E, F**). When the numbers of identified SARS-CoV-2-specific CD8^+^ T cells were analyzed, each participant showed varying numbers of identified SARS-CoV-2-specific CD8^+^ T cells but with a small waning over time (**Figure 4D**). For participant NH09 (A*01:01/A*32:01), there was a clear immunodominant response to epitope TTDPSFLGRY (ORF1ab), but also against three other epitopes to a lesser degree. While SARS-CoV-2-specific CD8^+^ T cells were detected against this epitope for participant NH52 (A*01:01/A*11:01), this participant had a much broader response against multiple SARS-CoV-2 epitopes of both HLA types (**Figure 4D**). For the other two participants (NH27 [A*02:01/A*03:01] and NH40 [A*02:01]), low frequencies of SARS-CoV-2-specific CD8^+^ T cells were detected. However, amongst the identified cells, the strongest response was detected against epitope YLQPRTFLL (S).

### 3.6 Characterization of antigen-specific T cell subsets using single-cell RNA sequencing

**Figure 5:**
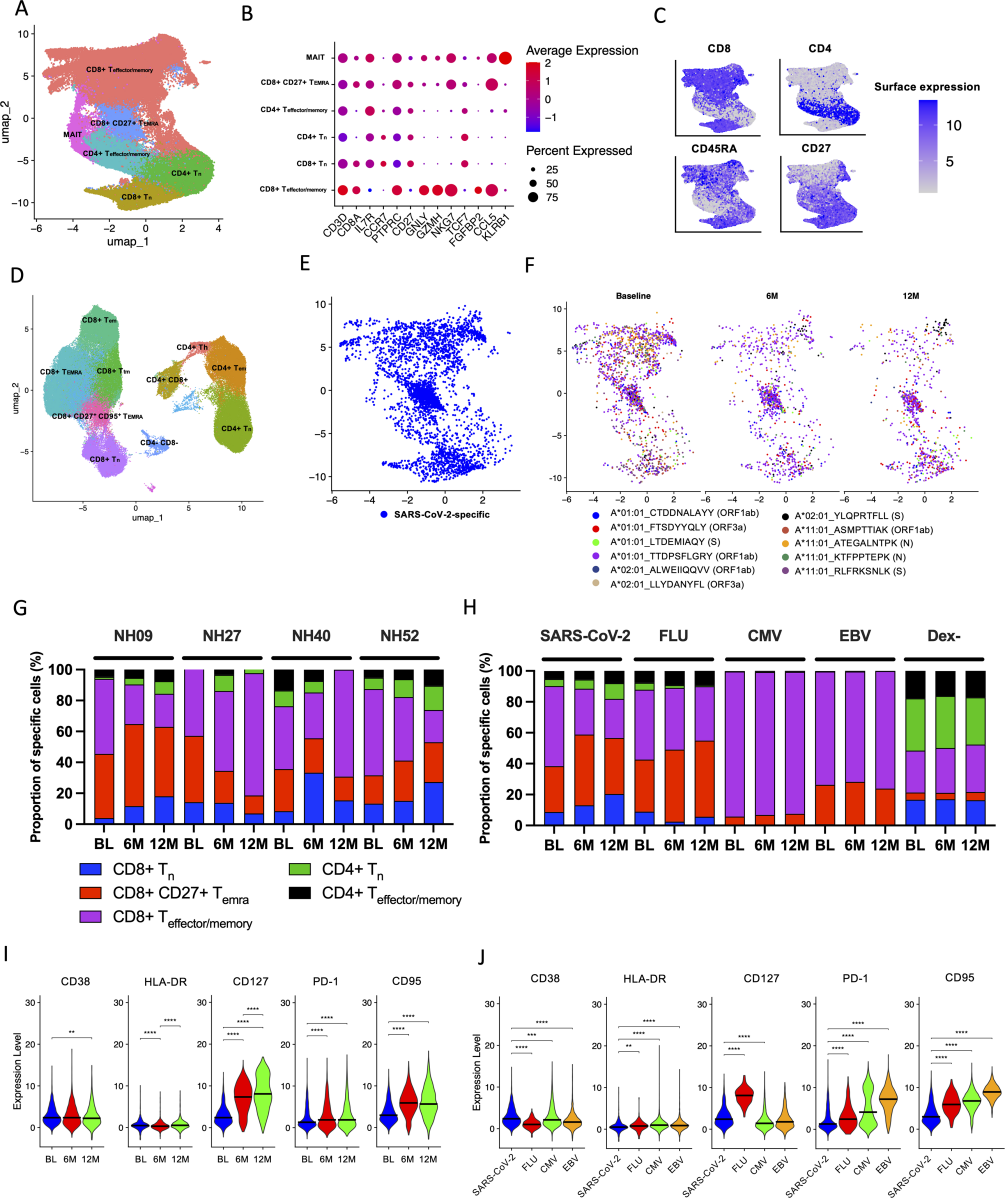
Characterization of the SARS-CoV-2-specific CD8^+^ T cell subsets over time. **(A)** Differentiation of the different T cell subsets via RNA-seq gene expression shown as a UMAP. Using RNA-seq gene expression alone, differentiation of CD8^+^ T_effector/memory_ (red), CD8^+^ CD27^+^ T_EMRA_ (blue), CD8^+^ T_n_ (yellow), CD4^+^ T_n_ (green), CD4^+^ T_effector/memory_ (teal) and MAIT (pink) cells was possible. Each dot represents a single cell. **(B)** Relative RNA-seq gene expression of the different differentially expressed genes (X axis) among the different T cell subsets (Y axis). The circle size represents the overall proportion of cells expressing that gene. A higher overall level of gene expression among the cell subsets is shown in red, while a lower overall gene expression is shown in blue. **(C)** Surface expression levels of bound CD4, CD8, CD45RA and CD27 CITE-seq antibodies amongst the different T cell subsets. A darker blue represents elevated surface expression of the indicated CITE-seq antibody. Each dot represents a single cell. **(D)** Differentiation of the different T cell subsets using CITE-seq antibodies. Each color represents a single T cell subset as indicated in the figure. Each dot represents a single cell. **(E)** UMAP showing the distribution of all identified SARS-CoV-2-specific CD8^+^ T cells (blue) using the same UMAP projection as shown in **(A)**. **(F)** UMAPs showing the distribution of epitope specificities of SARS-CoV-2-specific CD8^+^ T cells at the different time points using the same UMAP projection as shown in **(A)**. Each color represents a different epitope as indicated in the figure. **(G)** The proportion (%) of SARS-CoV-2-specific of T_n_ (blue), CD27^+^ T_EMRA_ (red) and T_effector/memory_ (purple) subsets for each individual participant at the BL, 6M and 12M time points. **(H)** The proportion (%) of each of the T cell subsets from **(G)** in T cells in the different identified antigen-specific CD8^+^ T cell populations, as well as Dextramer-negative (Dex-) cells for all four subjects at the BL, 6M and 12M time points. **(I)** Relative gene expression levels of CD38, HLA-DR, CD127, PD-1 and CD95 over the BL, 6M and 12M time points in all identified SARS-CoV-2-specific T cells from all participants. The black bar depicts the median. **(J)** Relative gene expression levels of CD38, HLA-DR, CD127, PD-1 and CD95 for all identified SARS-CoV-2-specific, flu-specific, CMV-specific, and EBV-specific T cells at the BL time point. The black bar depicts the median. Statistical analyses were performed using the Kruskal-Wallis test with Dunn’s multiple comparisons. Significant differences are shown as ^∗∗^ *p* < 0.01, ^∗∗∗^ *p* < 0.001, ^∗∗∗∗^ *p* < 0.0001.

To characterize identified antigen-specific T cells, clustering of all sorted T cells was first done using scRNAseq data paired with binding of CITE-seq antibodies to allow discrimination of the different T cell subsets. This allowed discrimination of CD8^+^ T cells into T_effector/memory_, T_n_ and CD27^+^ T_EMRA_ cells and CD4^+^ cells into T_n_ and T_effector/memory_ cells (**Figures 5A, B**). The T_effector/memory_ cells were a combination of T_em_, T_tm_ and T_EMRA_ cells that could not be discriminated by using the scRNAseq data alone. However, surface CITE antibodies allowed further discrimination into T_em_, T_tm,_ T_EMRA_, CD27+ T_EMRA_ and CD27^+^ CD95^+^ T_EMRA_ for CD8^+^ T cells and T_em_ and T helper (T_h_) cells for CD4^+^ T cells (**Figures 5C, D**; **Supplementary Figures S5B, C**). When all identified SARS-CoV-2-specific T cells from all participants and all time points were analyzed together, SARS-CoV-2-specific T cells were found to be widely distributed across the different T cell subsets with a high concentration among CD27^+^ T_EMRA_ and T_n_ cells (**Figure 5E**). It was also clear to see that SARS-CoV-2-specific T cells in the effector subsets waned over time, while the SARS-CoV-2-specific T cells in the CD27^+^ T_EMRA_ and T_n_ subsets appeared to be better maintained (**Figure 5F**). This became more evident when looking at the proportion of each T cell subset from all participants at each time point (**Figure 5G**). When compared to other antigen-specific T cells, the proportion of SARS-CoV-2-specific T cell subsets appeared to closely resemble that seen in flu-specific T cells but not CMV-specific or EBV-specific T cells (**Figure 5H**). When using the characterization of SARS-CoV-2-specific cells based on the surface CITE-seq antibodies, a similar decrease in the proportion of T_em_, T_tm_ and T_EMRA_ and especially an increase in the CD27^+^ CD95^+^ T_EMRA_ and T_n_ cells over time was found (**Supplementary Figures S5D–G**). The population was dominated by the CD27^+^ CD95^+^ T_EMRA_ cells that made up 50% at 6M and 12M time points (**Supplementary Figure S5F**). Looking at each participant individually, the proportions of SARS-CoV-2-specific T cell subsets generally followed a similar pattern over time, with the exception of participant NH40 who was found to have increasing proportions of effector T cell subsets (**Supplementary Figure S5G**). However, characterization using the surface CITE-seq antibodies revealed flu-specific T cells to have a major proportion of T_tm_ instead of T_n_ cells compared to SARS-CoV-2, while CMV-specific T cells were dominated by T_em_, T_tm_ and T_EMRA_ T cell subsets and EBV-specific T cells were dominated by CD27^+^ T_EMRA,_ T_tm_ and T_em_ T cell subsets (**Supplementary Figure S5F**).

When the expression of CD38, HLA-DR, CD127, PD-1 and CD95 was explored in the SARS-CoV-2-specific T cells over time, increasing expression levels of CD127 was found (**Figure 5I**; **Supplementary Figure S5A**). Compared to the BL time point, higher expression of PD-1 and CD95 was detected at both the 6M and 12M time points. By contrast, decreased expression of CD38 and HLA-DR were detected over time. When compared to other antigen-specific T cells (CEF-specific), SARS-CoV-2-specific T cells were found to have lower expression levels of CD95, PD-1 and HLA-DR (**Figure 5J**). By contrast, higher levels of expression of CD38 were detected in the SARS-CoV-2-specfic T cells when compared to the other antigen-specific T cells.

### 3.7 Identification of T cell receptor clonotypes using single-cell RNA sequencing

**Figure 6:**
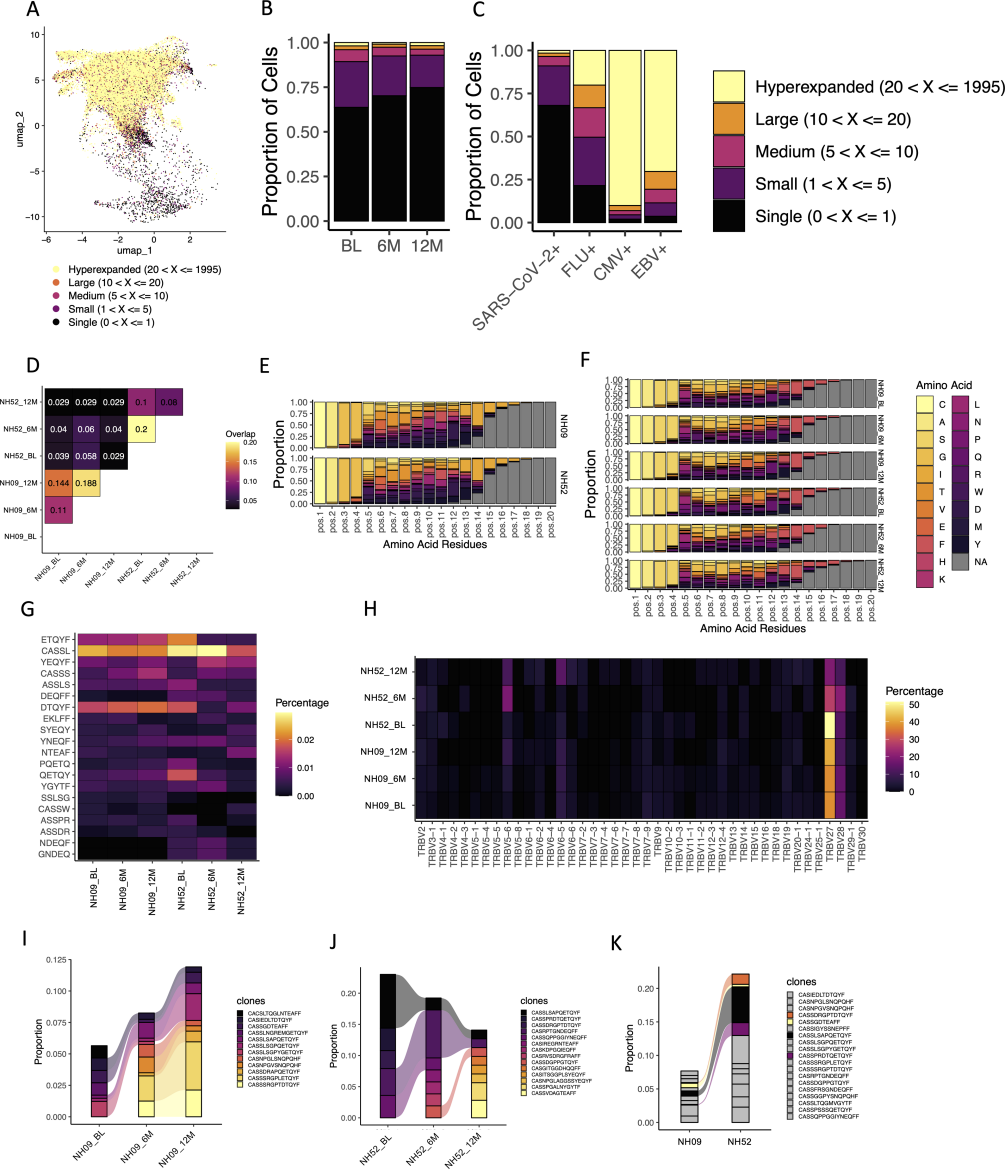
TCR usage amongst identified antigen-specific T cells. **(A)** The levels of TCR expansion amongst SARS-CoV-2-specific and CEF-specific T cells using the same clustering UMAP as shown in **Figure 5**. The different levels of expansion are indicated by the different colors in the figure. **(B)** The fractions of TCR expansion amongst SARS-CoV-2-specific T cells at the BL, 6M and 12M time points. Each color represents a different level of TCR expansion as indicated in the figure. **(C)** The fractions of TCR expansion amongst SARS-CoV-2-specific, flu-specific, CMV-specific and EBV-specific T cells from all time points. Each color represents a different level of TCR expansion as indicated in the figure. **(D)** The level of overlap found in the TRB-CDR3s of TTDPSFLGRY-specific T cells at the BL, 6M and 12M time points and between participants NH09 and NH52. Each number represents a fraction of the same TRB-CDR3s found amongst the total TRB-CDR3s for those two points. A yellow/orange color indicates a higher level of overlap, and a black/purple color represents a lower level of overlap. **(E)** The amino acid usage amongst TTDPSFLGRY-specific TRB-CDR3s found within all the time points (BL, 6M and 12M) for participants NH09 (top) and NH52 (bottom). Each color represents a different amino acid as indicated in the figure. Pos indicates the relative position of that amino acid in the TRB-CDR3. **(F)** The amino acid usage amongst TTDPSFLGRY-specific TRB-CDR3s at each individual time point for participants NH09 and NH52. Each color represents a different amino acid as indicated in the figure legend. Pos indicates the relative position of that amino acid in the TRB-CDR3. **(G)** Heat map showing a 5-k-mer analysis of the TRB-CDR3 amino acid sequences presenting the 20 top 5-mer usage shown as percentage (shown in the figure) at the rows and the subject timepoints shown in the columns (annotated below). **(H)** Heatmap of the TRBV gene usage in TTDPSFLGRY-specific T cells for participants NH09 and NH52 shown at the BL, 6M and 12M time points (shown in each row). Each column represents a TRBV gene and each row the subject timepoint with the color indicating the percentage of usage explained in the legend bar. **(I)** The fractions of the different identified TRB-CDR3s within TTDPSFLGRY-specific T cells for participant NH09 at the BL, 6M and 12M time points. Colors indicate the different TRB-CDR3 amino acid sequences as shown in the figure legend. Colors that are connected show identification of the same amino acid sequences at different time points. **(J)** The fractions of the different identified TRB-CDR3s within TTDPSFLGRY-specific T cells for participant NH52 at the BL, 6M and 12M time points. Colors indicate the different TRB-CDR3 amino acid sequences as shown in the figure legend. Colors that are connected show identification of the same amino acid sequences at different time points. **(K)** The average fractions of the different identified TRB-CDR3s within TTDPSFLGRY-specific T cells from all time points in participants NH09 and NH52. The grey color indicates TRB-CDR3 amino acid sequences that are not shared between the two participants. All other colors indicate TRB-CDR3 amino acid sequences that are shared between the two participants.

When looking at the total T cell population amongst all four participants at all time points, analysis of TCRs revealed that the highest level of expansion of clonotypes was in the CD8^+^ T_effector/memory_ cells, while lower levels of expansion were found in the other T cell subsets (**Figure 6A**). In the SARS-CoV-2-specific T cells, specifically, low levels of expansion were found amongst most of the identified clonotypes at all time points (**Figure 6B**). When compared to the other antigen-specific T cells, the level of expansion seen in the SARS-CoV-2-specific T cells most closely resembled that seen in the flu-specific T cell population (**Figure 6C**). The majority of CMV-specific and EBV-specific T cell populations was found to have hyperexpanded TCR repertoires (> 20 identical clones; **Figure 6C**).

Two of the participants (NH09 and NH52) had T cells specific to the TTDPSFLGRY (ORF1ab) epitope. When these T cells were compared, a substantial sequence overlap of the TCR beta chain (TRB)-complementary determining region 3 (CDR3) was found both across time and to a lesser degree between the two participants (**Figure 6D**). When looking at the specific amino acids within the TRB-CDR3 for the T cells specific to this epitope, the distribution was found to be almost identical between the two participants (**Figure 6E**). Similarly, this distribution was found to be conserved across time in both participants (**Figure 6F**). In support of this, a k-mer analysis of the TRB-CD3 aa sequences was performed, showing the 5-mer top motifs to be similar both between the two participants and across time (**Figure 6G**). Looking at the TRB gene usage in the TTDPSFLGRY-specific T cells, TRBV27 and TRBV28 were found to be the dominant gene used for both participants (**Figure 6H**). Within both participants (NH09 and NH52), the TRB-CDR3 sequences were found to be highly conserved across time (**Figures 6I, J**). In addition, 4 identical TRB-CHR3 sequences from TTDPSFLGRY-specific T cells were found between the two participants, indicating that there could be a shared repertoire (**Figure 6K**). The cells with overlapping TRB-CHR3 sequences were found to be CD45RA^+^ and focused between the T_EMRA_, CD27^+^ T_EMRA_ and T_n_ cells.

### 3.8 Identification of SARS-CoV-2 specific CD4^+^ T cells from single-cell RNA sequencing

**Figure 7:**
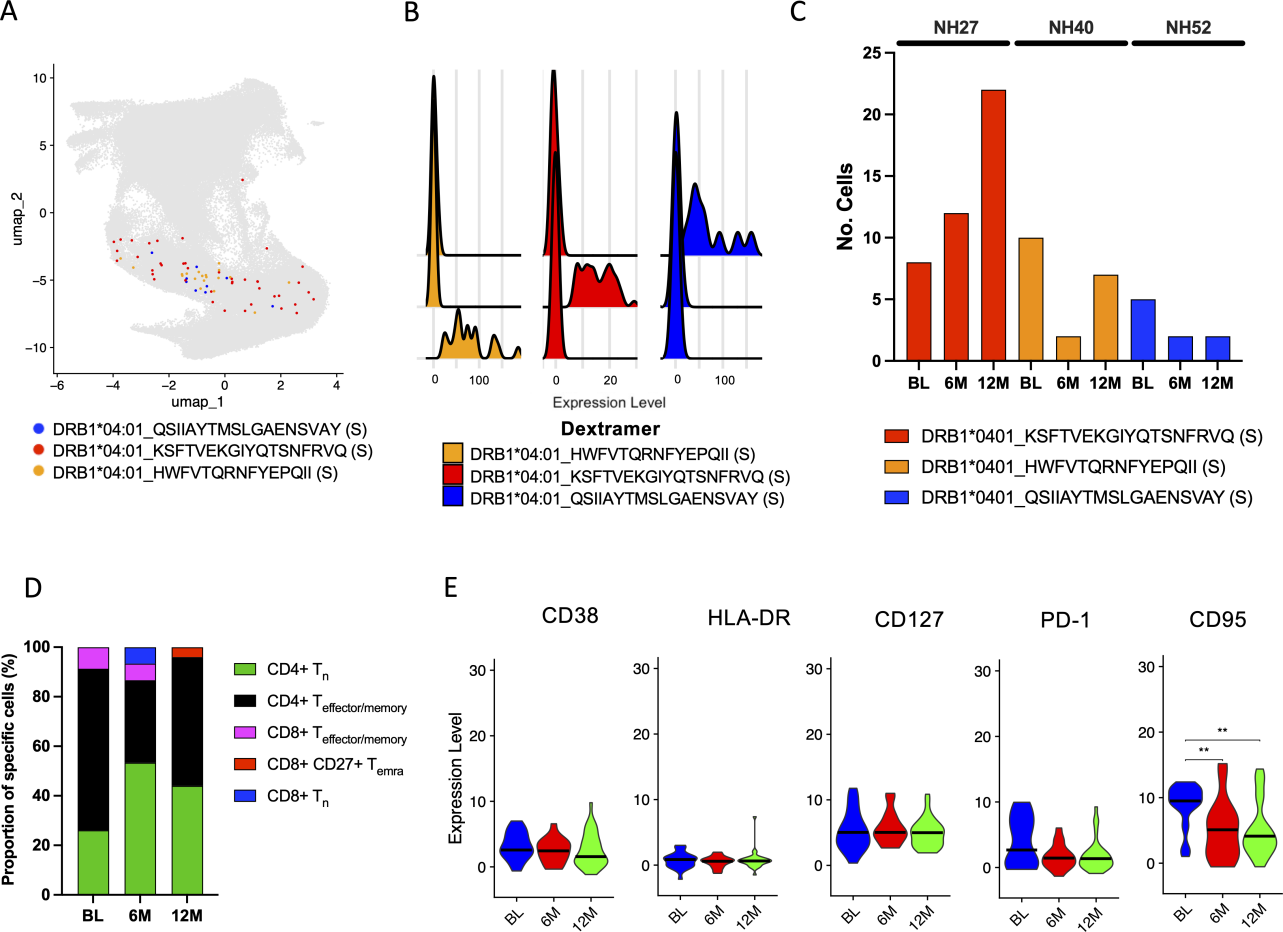
Identification and characterization of SARS-CoV-2-specific CD4^+^ T cells. **(A)** UMAP showing the distribution of identified SARS-CoV-2-specific CD4^+^ T cells using the same UMAP projection from **Figure 5**. Each color represents a different epitope specificity as indicated in the figure. Each dot represents a single cell. **(B)** Ridgeplot showing the specificity of the identified Dextramer-bound DRB1*04:01-specific CD4^+^ T cells. Each color represents a single epitope as indicated in the figure. The X axis represents the relative normalized binding of each Dextramer within each cluster, thus showing the specificity of the Dextramer signal within each cluster. **(C)** The total number of identified SARS-CoV-2-specific CD4^+^ T cells identified compared to total CD4^+^/CD8^-^ for each participant at each time point. **(D)** The proportion (%) of MHC class II Dextramer-bound CD4^+^ T_n_ (green), CD4^+^ T_effector/memory_ (black), CD8^+^ T_effector/memory_ (pink), CD8^+^ CD27^+^ T_EMRA_ (red), CD8^+^ T_n_ (blue) T cell subsets at the BL, 6M and 12M time points for all participants. **(E)** The relative expression of CD38, HLA-DR, CD127, PD-1 and CD95 in MHC class II Dextramer-bound T cells at the BL, 6M and 12M time points for all participants. The black bar depicts the median. Statistics were done using Kruskal-Wallis tests with adjustment for multiple comparisons using Dunn’s test. Significant differences are shown as ^∗∗^ *p* < 0.01.

In an attempt to identify SARS-CoV-2-specific CD4^+^ T cells, dCODE Dextramer^®^ reagents loaded with MHC class II epitopes specific to SARS-CoV-2 (**Supplementary Table S5**) were added during cell sorting of the 4 participants (NH09, NH27, NH40 and NH52). Following this, scRNAseq using the Chromium platform was used to identify antigen-specific cells. Compared to the number of antigen-specific cells identified for the MHC class I epitopes, the number of SARS-CoV-2-specific CD4^+^ T cells identified was low. However, deconvolution of the epitope specificities revealed 3 epitopes within the S protein restricted to DRB1*04:01 (**Figures 7A, B**). These were identified in small numbers from participants NH27, NH40 and NH52 but none were shared between the participants (**Figure 7C**). Unlike the identified MHC class I epitopes, which were found to be widely spread amongst T cell subsets, the identified Dextramer-bound T cells here were mostly found to cluster within the CD4^+^ T cell subsets (**Figure 7D**). More specifically, the proportion of identified Dextramer-bound T cells were found to be evenly distributed between T_n_ and T_effector/memory_ cell types, which appeared to remain consistent across the time points (**Figure 7D**). Further dissemination by the surface CITE antibodies showed the T_em_ cells to be dominant, with smaller proportions of T_n_, T_h_ and the T_cm_ subsets, which were sustained over time (**Supplementary Figure S6A**). Analysis of expression of CD38, HLA-DR, CD127, PD-1 and CD95 within the Dextramer-bound T cells revealed a decrease in CD95 expression over time, while CD38, PD-1, HLA-DR and CD127 expression was found to be similar across the different time points (**Figure 7E**). Although the frequency and number of Dextramer-bound CD4^+^ T cells was small, an expansion of TCR clonotypes for participant NH40 and NH52 was observed (**Supplementary Figure S6B**), but mainly at baseline (**Supplementary Figure S6C**). An overlap in the TRB-CDR3 sequence for participant NH40 and NH52 was found, but there were different CDR3 sequence distributions at each position between all three participants (**Supplementary Figures S6D, E**). Further dissemination by k-mer analysis revealed distinct patterns within the CDR3 aa sequence for NH40 and NH52 being dominated by a few k-mers (**Supplementary Figure S6F**). In addition, TRB gene usage was dominated by TRBV27 and TRBV6-2 for participant NH52 and participant NH40, respectively, while participant NH27 had varied gene usage (**Supplementary Figure S6G**). Finally, a level of overlapping TRB-CDR3 receptor usage was found within participants NH40 and NH52 at baseline compared to 12 months showing high conservation, while no overlap was found for NH27 (**Supplementary Figures S6H-J**).

## 4 Discussion

Unravelling immune responses after infection is essential for the understanding of protective immunity as well as vaccine design. In this study, scRNAseq of antigen-specific T cells revealed shared TRB-CDR3 regions between individuals with non-hospitalized primary SARS-CoV-2 infection in CD8^+^ T cells as well as identification of SARS-CoV-2-specific CD4^+^ T cells. Longitudinal identification, and characterization, of SARS-CoV-2-specific CD8^+^ T cells after a primary infection event showed that, although the overall frequencies of these T cells declined over time, some were found to persist up to 12M post-symptom onset, which aligns with findings from other studies (43, 44). This observed decline in the SARS-CoV-2 CD8^+^ T cell frequencies is likely a result of the contraction phase following infection, whereby the excess antigen-specific cells undergo apoptosis (45). Compared to our previous study assessing SARS-CoV-2-specific CD8^+^ T cells following serial mRNA vaccinations (up to 4 Comirnaty^®^ doses), the frequencies of detected antigen-specific CD8^+^ T cells were highly comparable to the frequencies found in this study (34). Compared to the stable frequencies of CEF-specific CD8^+^ T cells, SARS-CoV-2-specific CD8^+^ T cell frequencies were lower, likely due to the different disease states induced by these infections. EBV and CMV infections, which cause life-long latent infections that can reactivate throughout one’s lifetime (46, 47), also showed different proportions of T cell status. While CEF-specific CD8^+^ T cells were found to have higher proportions of T_tm_ and T_em_ T cell subsets, which are more mature states of CD8^+^ T cells, SARS-CoV-2-specific CD8^+^ T cells were found to have higher proportions of CD27^+^ T_EMRA_ and T_n_ cells, indicating more naïve states of CD8^+^ T cells. Although influenza infections are similar to SARS-CoV-2 infections as respiratory infections with similar mortality rates (48), flu-specific CD8^+^ T cells could not be discriminated from CMV-specific and EBV-specific CD8^+^ T cells in flow cytometry due to the experimental setup. However, scRNAseq of flu-specific CD8^+^ T cells revealed that they share a similar T cell subset profile to SARS-CoV-2-specific CD8^+^ T cells, suggesting similarities between these different antigen-specific cells.

Interestingly, it was found that the proportion of T_n_ cells within the SARS-CoV-2-specific CD8^+^ T cell populations increased over time, a finding also observed by others (27). More specifically, the enrichment of T_n_ cells noted by others showed that these cells were identified as stem cell-like memory T (T_SCM_) cells, which share characteristics with T_n_ cells but have high expression of CD95 and CD127 (49). Notably, these cells have been reported to be found following both COVID-19 vaccination (50, 51) and SARS-CoV-2 infection (27, 43). Although T_SCM_ cells were not directly measured in the present study, scRNAseq revealed an increase in CD95 expression in SARS-CoV-2-specifc CD8^+^ T cells over time, suggesting that the observed enrichment of T_n_ cells could be T_SCM_ cells. Furthermore, both flow cytometry and scRNAseq showed high CD127 expression among SARS-CoV-2-specific CD8^+^ T cells. In addition, scRNAseq CITE-seq clustering analysis revealed that the majority these SARS-CoV-2-specific cells have high expression of CD95 as well as CD127. This phenotype increased from BL to 6M and was further sustained to 12M. When comparing SARS-CoV-2 positive cells to the CEF Dextramer positive cells, CD27^+^ CD95^+^ T_EMRA_ cells was found at much higher frequency, providing further evidence that the enrichment of CD27^+^ CD95^+^ T_EMRA_ and T_n_ naïve-like cells found by CITE-seq could be T_SCM_ cells and not just naïve cells. T_SCM_ cells are known for their capacity for homeostatic proliferation and multipotency, reconstituting both effector and memory T cell subsets upon antigen re-exposure (52). Recent studies on T_SCM_ cells after yellow fever vaccination showed these cells persist at stable levels for decades (53), possibly contributing to life-long protection (54). This suggests that T_SCM_ cells may play a central role in providing long-term protective immunity. Therefore, the induction of these T cells may be critical for long-term protection against COVID-19.

Deconvolution of the epitope specificities using scRNAseq showed that, while immunodominant SARS-CoV-2 epitopes exist for some, others may have a more heterogeneous response. More specifically, the A*01:01 restricted epitope TTDPSFLGRY targeting the ORF1a region was found to be immunodominant, which has also been reported by others (25, 26). However, it should be noted that deconvolution of SARS-CoV-2 epitopes was conducted in only four heterogenous individuals (each with different HLA types), which is a limitation for exploring immunodominant epitopes. Furthermore, the frequencies of identified SARS-CoV-2-specific CD8^+^ T cells were relatively low in two of these individuals. Interestingly, however, all epitope-specific T cells declined at similar rates, suggesting that longer-lived T cells are not epitope-specific for SARS-CoV-2. Importantly, most of these epitopes are conserved amongst the different SARS-CoV-2 variants (16), which will likely be important in re-exposure to the virus.

Utilizing scRNAseq, the TCRs of SARS-CoV-2-specific CD8^+^ T cells were found to be, generally, quite diverse, which has also been reported by others (55). Interestingly, T cells specific to TTDPSFLGRY were found to have highly shared TRB-CDR3 sequences between two individuals, which suggests a public TCR repertoire against this epitope. While others have alluded to shared SARS-CoV-2-specific TCR sequences between donors (56, 57), others have shown a high TCR repertoire diversity among SARS-CoV-2-specific CD8^+^ T cells (55). As the TTDPSFLGRY epitope is reported to be quite immunodominant among HLA A*01:01 individuals that have had a SARS-CoV-2 infection (25), it is perhaps not surprising that highly similar TRB-CDR3 sequences can be found between donors. In a recent study exploring immunodominant T cell responses to CMV, EBV and adenovirus, a large frequency of the virus-specific TCR sequences were found to be shared between donors (58). It was proposed that the high level of conservation of TCR sequences between donors is a result of T cells that were successful at controlling latent viruses. Although SARS-CoV-2 does not cause a latent infection, it may be possible that the TCR sequences found to be shared between individuals could be important for protection against SARS-CoV-2 infection and thus warrants further exploration. Additionally, the overall TCR gene usage between this study and others appears to be similar (55). Importantly, in this study, identical TRB-CDR3 were found across different time points in different individuals, indicating that these T cell clones were maintained over time but a succession of the TRB-CDR3 from BL to 6M was seen within subjects. The overall level of expansion of the SARS-CoV-2-specific TCRs was found to be low and similar to what we observed in the flu-specific TCRs. By contrast, CMV-specific and EBV-specific TCRs were found to be mostly hyperexpanded, which may be a result of the different disease states induced by these viruses, or that there may have been multiple virus reactivation episodes in these individuals, allowing for greater expansion of antigen-specific T cells. It is therefore possible that further antigen exposure, either via COVID-19 vaccination or reinfection, will drive TCR expansion in the SARS-CoV-2-specific CD8^+^ T cells.

Phenotypical characterization of SARS-CoV-2-specific CD8^+^ T cells using both flow cytometry and scRNAseq showed that, at the BL time point, SARS-CoV-2-specific CD8^+^ T cells presented with a more activated phenotype, expressing higher levels of HLA-DR, PD-1 and CD38 than at the later time points, where high expression of these markers was not observed. This finding is in accordance with other reports (59, 60), as this shift away from an activated phenotype after viral clearance is common following clearance of other acute viral infections (54, 61). This loss of an activated phenotype likely corresponds to the loss of T_EMRA_ cells over time, which are more cytotoxic and less proliferative than other T cell subsets (62, 63). However, as discussed above, expression of CD127 was found to be high and maintained over time in SARS-CoV-2-specific CD8^+^ T cells. While CD127 has been associated with activation of T cells (64), it has also been associated with memory and homeostatic proliferation (64). Given the increase in proportions of T_n_ cells over time, this high expression of CD127 is likely attributed to maintenance of SARS-CoV-2-specific T_SCM_ cells as shown by others (43). This is further supported by the loss of T_cm_ cells, which have also been reported to be long-lasting T cell subsets with a high proliferation potential (65, 66). Interestingly, small populations of T_EMRA_ cells expressing CD27 were found, which were maintained over time for SARS-CoV-2-specific CD8^+^ T cells as well as CEF-specific CD8^+^ T cells. While the role of this T cell subset is largely unknown, these could simply be cells transitioning into T_EMRA_ cells by gradual loss of CD27 (62) or into memory cells by gradual loss of CD45RA (67). However, given that this T cell subset was also found in high frequency within the flu-specific CD8^+^ T cell population, they could play a crucial role in acute respiratory viral infections that has yet to be explored.

Using pools of MHC class II Dextramer reagents, scRNAseq identified small populations of SARS-CoV-2-specific CD4^+^ cells in three individuals. Deconvolution of the epitopes showed they were all directed towards the S protein and all restricted to DRB1*04:01. While epitopes restricted to DRB1*07:01 and DRB1*15:01 were also investigated, no antigen-specific CD4^+^ T cells to these epitopes were found. However, it is important to note that the overall frequencies of these epitope-specific CD4^+^ T cells were low, and analysis of these cells required pooling of all 3 time points (BL, 6M and 12M). In contrast to SARS-CoV-2-specific CD8^+^ cells, the SARS-CoV-2-specific CD4^+^ T cell subsets were dominated by T_em_ cells, which were maintained until the 12M time point. Further, this T cell subset had TCR overlap and recall over time found in the TRB-CDR3 sequences within two of the epitopes. Analysis of CD38, HLA-DR, CD127 and PD-1 expression in the SARS-CoV-2-specific CD4^+^ cells showed no changes over time, reflecting the stability of the CD4^+^ T cells subsets. Unlike the increased CD95 expression seen in SARS-CoV-2-specific CD8^+^ T cells over time, CD95 expression in the SARS-CoV-2-specific CD4^+^ T cells decreased over time. While elevated CD95 expression in T cells is common following various viral infections (68–70), the role of CD95 in CD4^+^ T cells in early infection appears to be associated with activation (71), whereas increased expression of CD95 is associated with disease progression in HIV infection (72). Therefore, the decrease in CD95 expression in the SARS-CoV-2-specific CD4^+^ T cells likely reflect decreasing levels of activation.

This study has some limitations. One key limitation is the sample size, particularly for the 12M time point and for the scRNAseq analysis. Excluding vaccinated individuals at the 12M time point has also limited the chance to explore what happens to the antigen-specific T cells upon antigen re-exposure, particularly those specific to the spike protein. Within the scRNAseq data, low numbers of antigen-specific cells were recovered from two of the four individuals, meaning that the overall analysis of SARS-CoV-2-specific T cells was skewed to the two individuals that had higher cell numbers. Another limitation is that this study had no other group to compare to, whether that be hospitalized SARS-CoV-2 infections or healthy individuals, meaning that comparisons could only be done within the same group.

In summary, this study found long-lasting SARS-CoV-2-specific CD8^+^ T cell responses primarily composed of T_n_ cells, which were more likely to be T_SCM_ cells due to high CD95 and CD127 expression. Deconvolution of epitope specificities revealed that, while some individuals may have a CD8^+^ T cell response dominated by a few epitopes, others may have a more heterogeneous CD8^+^ T cell response, possibly depending on HLA restriction. SARS-CoV-2-specific CD8^+^ TCRs were generally found to be diverse with low levels of expansion, however, TCRs targeting the same epitope in two different individuals shared clonality, suggesting a public TCR repertoire for specific epitopes. Finally, although SARS-CoV-2-specific CD4^+^ T cells proved more difficult to identify, they exhibited decreasing levels of CD95 expression and were mainly composed of effector memory cells, indicating reduced activation but stable memory levels over time.

## Data Availability

Following publication, and in agreement with the Data Protection Agency, Denmark, the data generated in this study will be made available to researchers who provide a sound proposal. Proposals should be directed to jbukh@sund.ku.dk, and to gain access, data requestors will need to sign a data access agreement. Individual participant data will remain coded.
