# Supplementary material for: T cell receptor usage and epitope specificity amongst CD8+ and CD4+ SARS-CoV-2-specific T cells
Source: Front Immunol. 2025 Feb 28;16:1510436. doi: 10.3389/fimmu.2025.1510436 (PMC11906682; doi:10.3389/fimmu.2025.1510436)
Supplement: Supplementary file 1 [file DataSheet1.pdf]

Supplementary Material

# 1 Supplementary Figures and Tables

## 1.1 Supplementary Figures

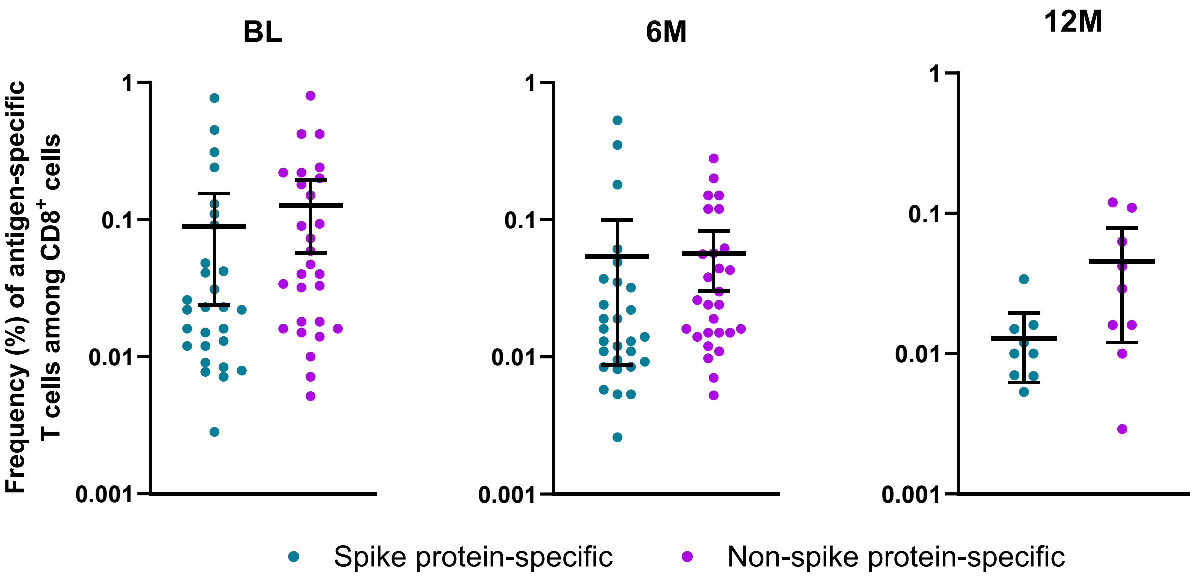


**Supplementary Figure 1.** Comparison of $CD8^+$ T cell frequencies of SARS-CoV-2 spike protein-specific (green) and non-spike protein-specific (purple) at the baseline (BL), 6-month (6M) and 12M time points.

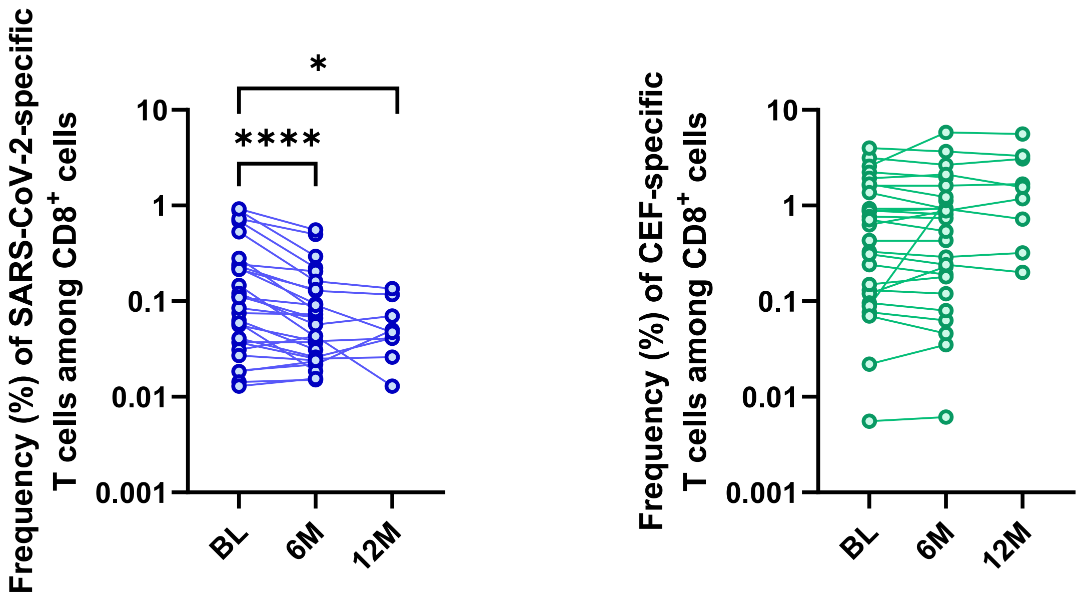


**Supplementary Figure 2.** Comparison of SARS-CoV-2-specific (left, blue) and CEF-specific (right, green) $CD8^+$ T cell frequencies at the baseline (BL), 6-month (6M) and 12M time points. Statistics were done using the Kruskal-Wallis test and corrected for multiple comparisons using Dunn's test. *$p$<0.05, ****$p$<0.0001

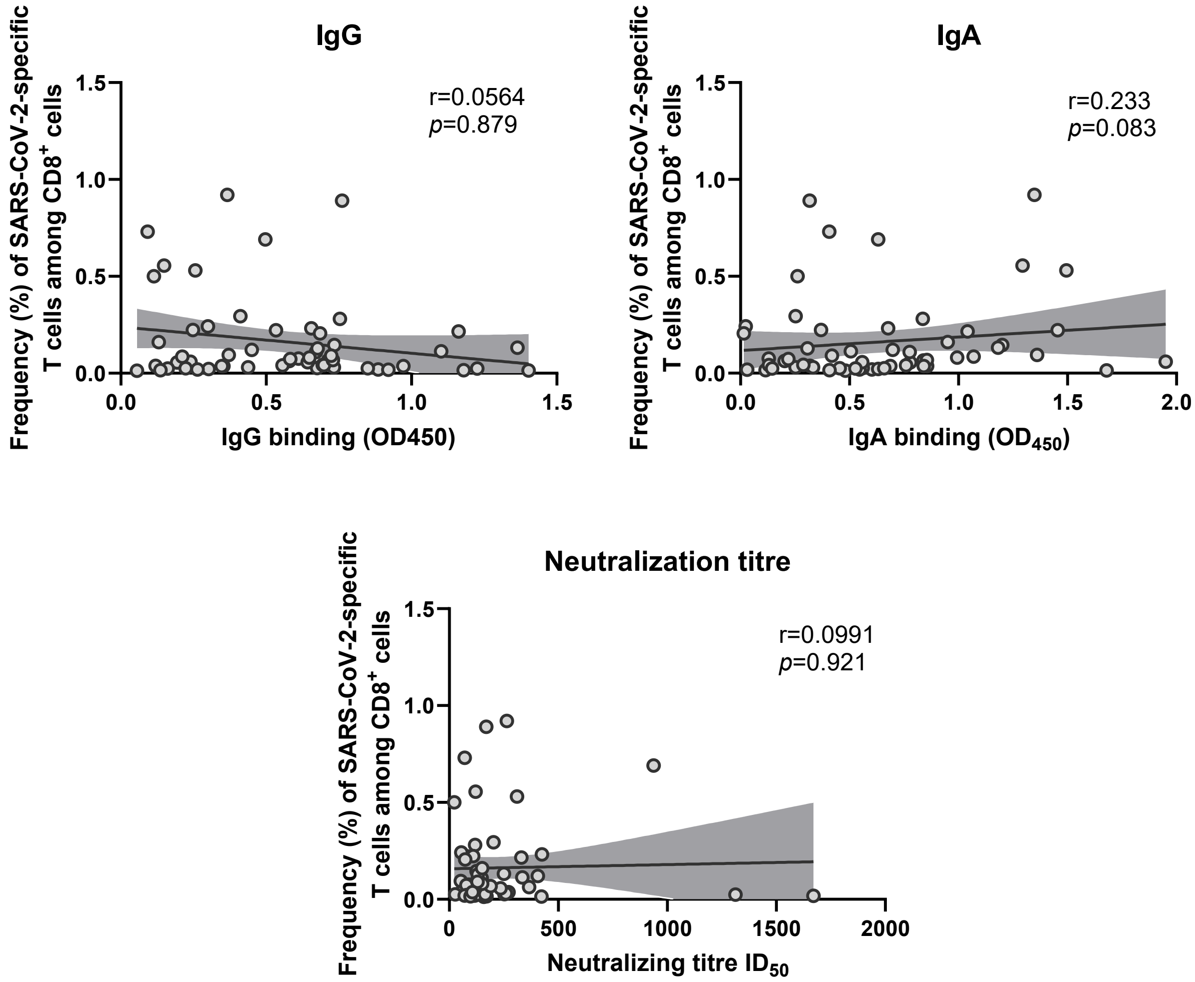


**Supplementary Figure 3.** Linear regression analyses of SARS-CoV-2-specific CD8$^+$ T cell frequencies and spike protein-specific IgG (top left), IgA (top right) and neutralizing titers to a D614G SARS-CoV-2 isolate (bottom) at the baseline and 6M time points. No significant correlations between CD8$^+$ T cell frequencies and IgG, IgA or neutralizing antibody levels were found.

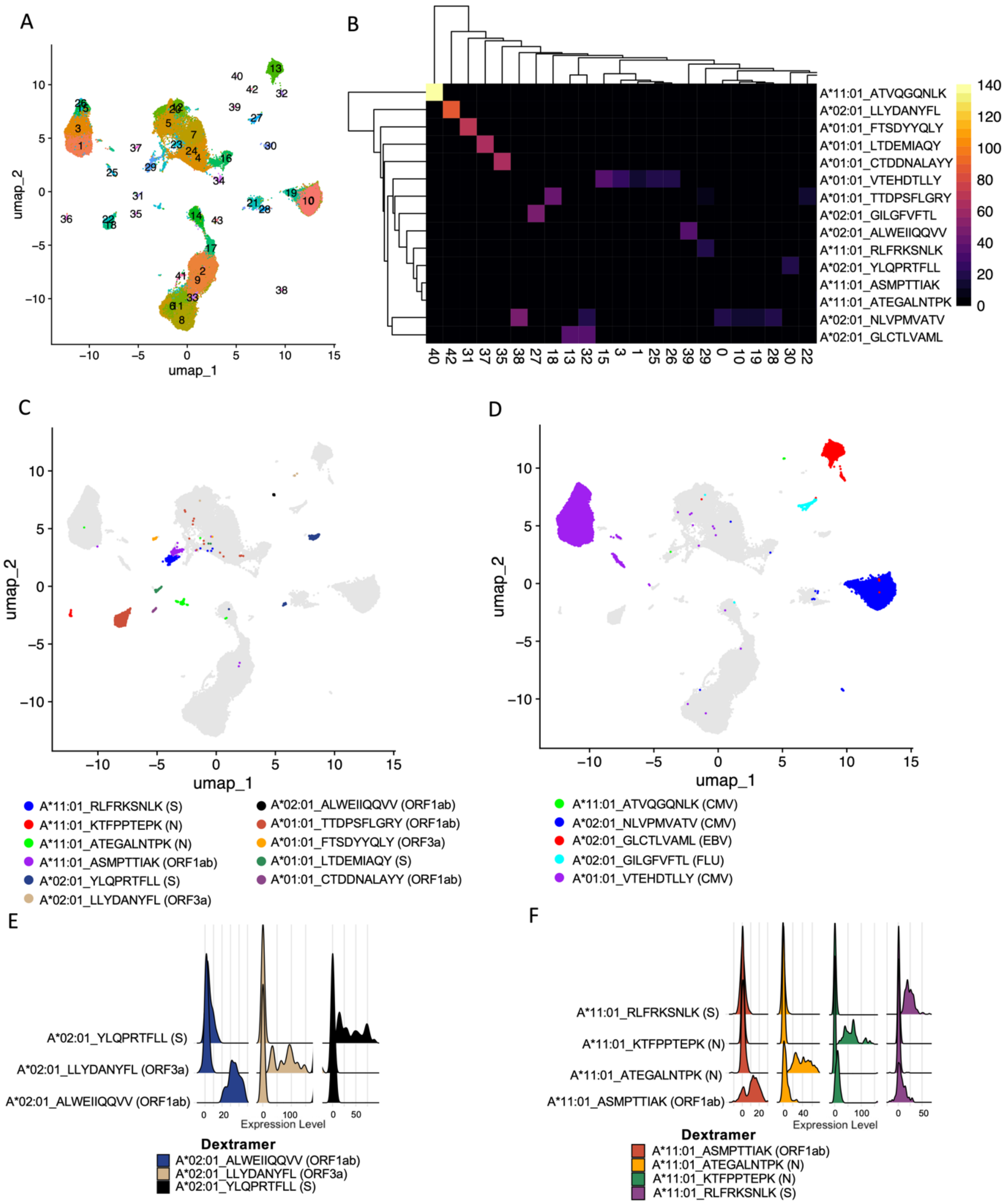


**Supplementary Figure 4. SARS-CoV-2- and CEF-specific CD8$^{+}$ T cells response defined by scRNAseq. A)** Antigen specific cells for all four subjects shown as surface CITE-seq UMAP including dextramers and surface antibodies after normalization of clusters. Numbering is shown centered of

each cluster. **B)** Characterization of positive SARS-CoV-2- and CEF-specific clusters found in A shown as CITE-seq heatmap with columns representing clusters and rows showing antigen specificity. **C)** CITE-seq UMAP annotated by B with SARS-CoV-2 specific cells shown as colored dots as explained in the figure. **D)** CITE-seq UMAP annotated by B with CEF specific cells shown as colored dots explained in the figure. **E)** Ridgeplot showing the specificity of the identified Dextramer-bound SARS-CoV-2 A*02:01-specific $CD8^+$ T cells for participant NH27 and NH40. Each color represents a single MHC class I Dextramer as indicated in the figure. The X axis represents the relative normalized binding of each Dextramer within each cluster identified in A, showing the specificity of the Dextramer signal of each cluster of SARS-CoV-2 specific cell to the A*02:01 epitopes. **F)** Ridgeplot showing the specificity of the identified Dextramer-bound SARS-CoV-2 A*11:01-specific $CD8^+$ T cells for participant NH52. Each color represents a single MHC class I Dextramer as indicated in the figure legend. The X axis represents the relative normalized binding of each Dextramer within each cluster identified in A, showing the specificity of the Dextramer signal of each cluster of SARS-CoV-2 specific cell to the A*11:01 epitopes.

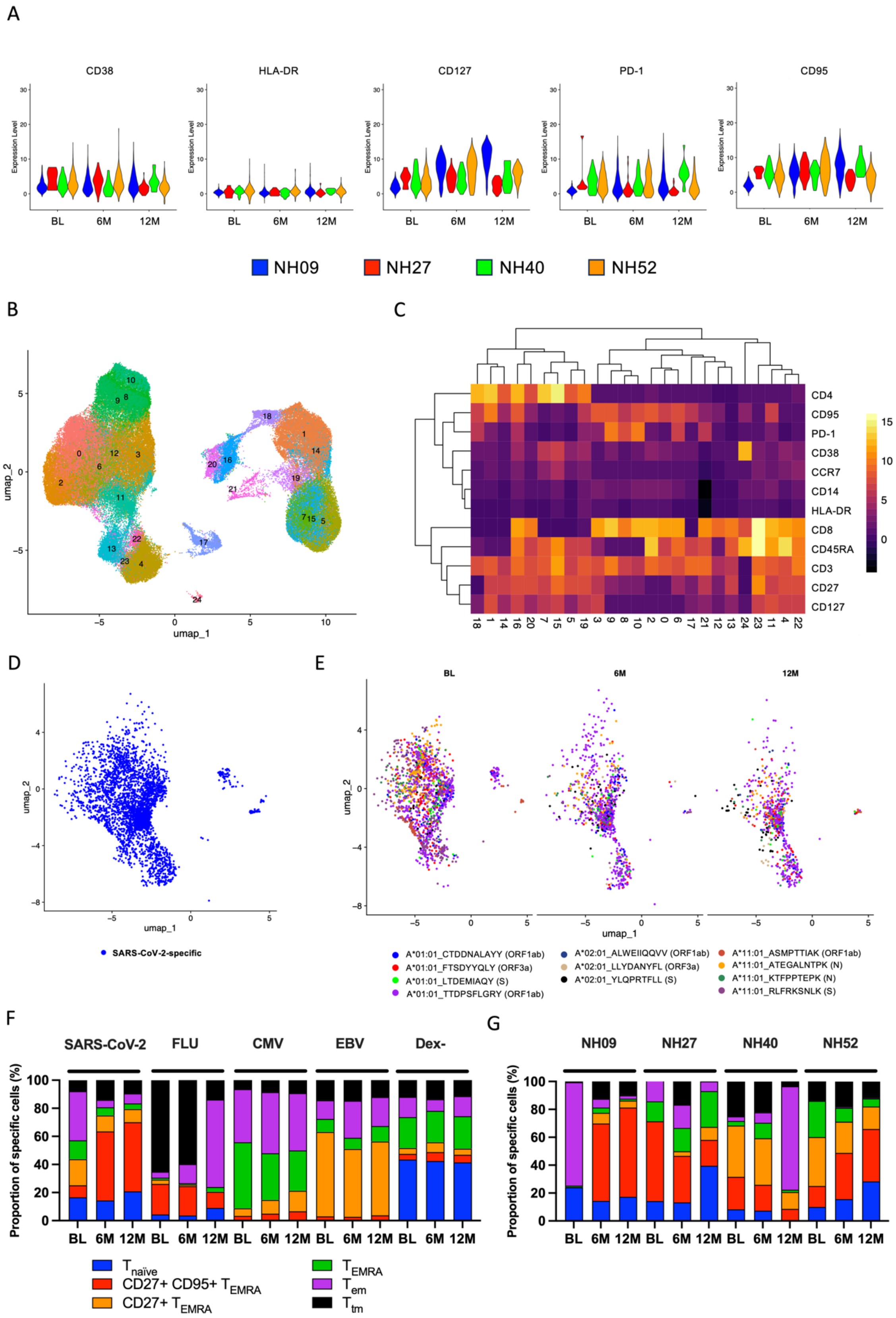
A
CD38
HLA-DR
CD127
PD-1
CD95
Expression Level
BL
6M
12M
NH09
NH27
NH40
NH52
B
umap_1
umap_2
C
CD4
CD95
PD-1
CD38
CCR7
CD14
HLA-DR
CD8
CD45RA
CD3
CD27
CD127
D
SARS-CoV-2-specific
E
BL
6M
12M
A*01:01_CTDDNALAYY (ORF1ab)
A*01:01_FTSDYYQLY (ORF3a)
A*01:01_LTDEMIAQY (S)
A*01:01_TTDPSFLGRY (ORF1ab)
A*02:01_ALWEIIQQVV (ORF1ab)
A*02:01_LLYDANYFL (ORF3a)
A*02:01_YLQPRTFLL (S)
A*11:01_ASMPTTIAK (ORF1ab)
A*11:01_ATEGALNTPK (N)
A*11:01_KTFPPTEPK (N)
A*11:01_RLFRKSNLK (S)
F
SARS-CoV-2
FLU
CMV
EBV
Dex-
Proportion of specific cells (%)
G
NH09
NH27
NH40
NH52
T naïve
CD27+ CD95+ T EMRA
CD27+ T EMRA
T EMRA
T em
T tm

**Supplementary Figure 5. SARS-CoV-2- CD8$^+$ T cells response defined by CITE-seq. A**) Relative gene expression levels of CD38, HLA-DR, CD127, PD-1 and CD95 over the BL, 6M and 12M time points in all identified SARS-CoV-2-specific T cells split in individual participants. **B)** Antigen specific cells for all four subjects shown as surface CITE-seq UMAP including only surface antibodies after normalization of clusters. Numbering is shown centered of each cluster. **C)** Characterization of positive SARS-CoV-2- and CEF-specific clusters found in A shown as CITE-seq heatmap with columns representing clusters and rows showing antigen specificity. **D)** CITE-seq UMAP showing the distribution of all identified SARS-CoV-2-specific CD8$^+$ T cells (blue) using the same UMAP projection as shown in **B**. **E)** CITE-seq UMAPs showing the distribution of epitope specificities of SARS-CoV-2-specific CD8$^+$ T cells at the different time points using the same UMAP projection as shown in **B**. Each color represents a different epitope as indicated in the figure. **F**) The proportion (%) of subsets defined by CITE-seq in the different identified antigen-specific CD8$^+$ T cell populations, as well as Dextramer-negative (Dex-) cells for all four subjects at the BL, 6M and 12M time points. **G)** SARS-CoV-2-specific CD8+ T cell differentiation based on surface CITE-seq for each of the three timepoints shown for each individual subject.

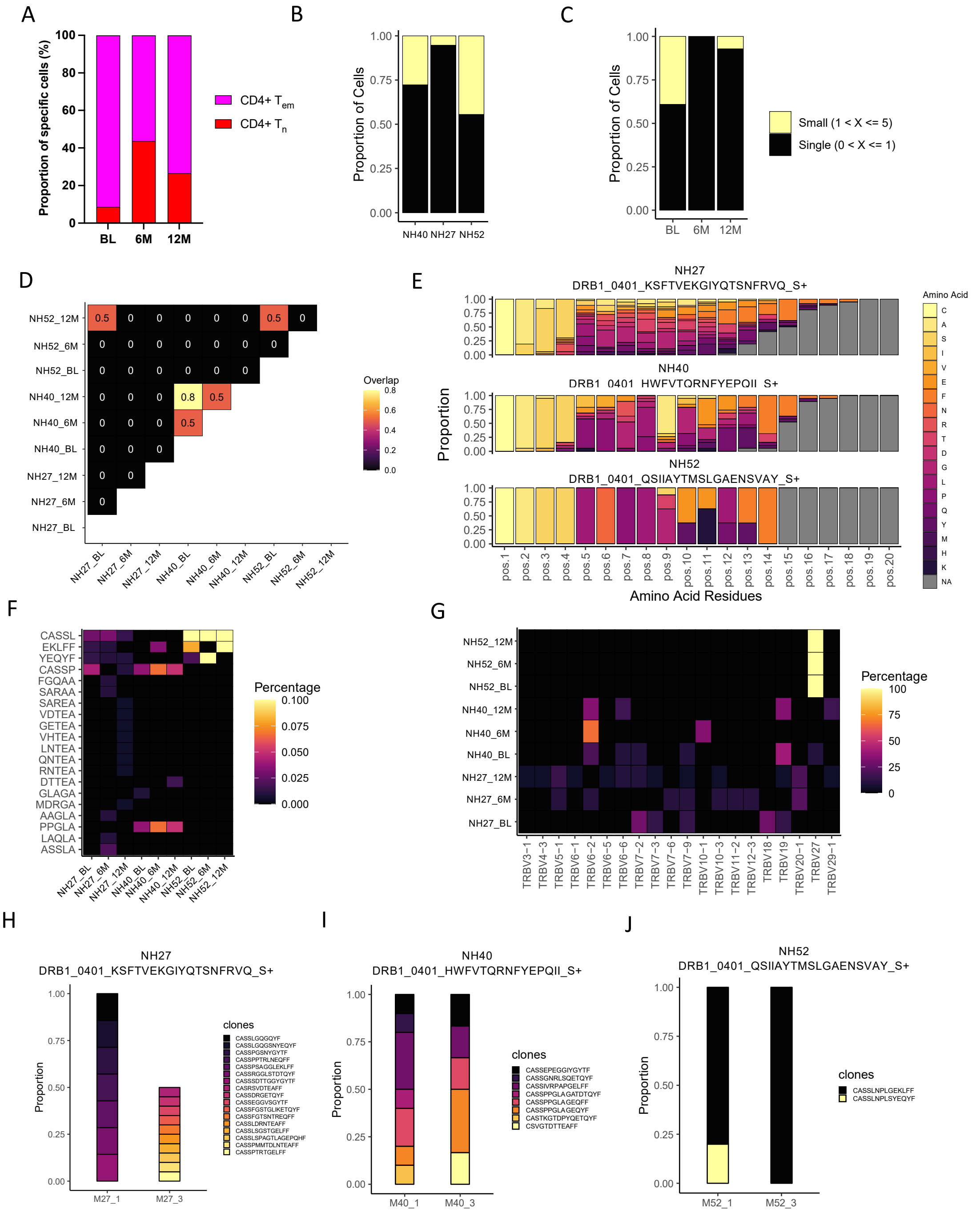


**Supplementary Figure 6. SARS-CoV-2-specific CD4$^+$ T cells differentiation and TCR usage A)** SARS-CoV-2-specific CD4+ T cell differentiation based on surface CITE antibodies CD45RA, CCR7 and CD27 measured for each of the three timepoints. **B)** Proportion of SARS-CoV-2-specific CD4$^+$ TCR expansion measured for each of the three participants shown as stacked bar graphs. Y-axis show

the proportion of cell expansion. **C)** SARS-CoV-2-specific $CD4^{+}$ TCR expansion from all three timepoints shown as stacked histograms. Y-axis show the proportion of cells expansion. **D)** Overlap map between the three subjects (NH27, NH40 and NH52) comparing all three timepoints looking at the TRB aa CDR3 sequence for CD4+ specific cells. **E) A)** Proportion at each position within the CDR3 sequence shown as stacked bars representing the aa distribution for the three subjects (NH27, NH40 and NH52). F) Heat map showing 5-mer aa k-mer analysis of the CDR3 sequence presenting the 20 top 5-mer results at the y-axis and the subject timepoints shown at the x-axis. **G)** Heatmap of the TRBV gene usage for the three subject timepoints shown at the y-axis and TRBV genes at the x-axis. **H)** Alluvial plots showing CDR3 aa sequences proportion as stacked bars with connecting BL and 12M within subject NH27. **I)** Alluvial plots showing CDR3 aa sequences proportion as stacked bars with connecting BL and 12M within subject NH40. **J)** Alluvial plots showing CDR3 aa sequences proportion as stacked bars with connecting BL and 12M within subject NH452.

## 1.2 Supplementary Tables

| Subject ID | Infecting variant identified (clade) | HLA Typing | | | | Baseline | | CD8+ T-cells (% of total) | | | 6 Months | | CD8+ T-cells (% of total) | | | 12 Months | | CD8+ T-cells (% of total) | | |
|---|---|---|---|---|---|---|---|---|---|---|---|---|---|---|---|---|---|---|---|---|
| | | A | | B | | Days post onset | ID50 | Spike | Non-spike | Total | Days post onset | ID50 | Spike | Non-spike | Total | Days post onset | ID50 | Spike | Non-spike | Total |
| NH07 | | 01.01.01 | 02.01.01 | 08.01.01 | 18.01.01 | 47 | 107 | 0.0084 | 0.0100 | 0.0184 | 201 | 89 | 0.0095 | 0.0140 | 0.0235 | 379 | 18 | 0.0120 | 0.0290 | 0.0410 |
| NH09 | | 01.01.01 | 32.01.01 | 08.01.01 | 40.02.01 | 19 | 425 | 0.0230 | 0.0140 | 0.0370 | 179 | 131 | 0.0220 | 0.0150 | 0.0370 | 377 | 28 | 0.0069 | 0.1100 | 0.1169 |
| NH13 | | 02.01.01 | 11.01.01 | 35.01.01 | 52.01.01 | 42 | 366 | 0.0160 | 0.0400 | 0.0560 | 191 | 259 | 0.0081 | 0.0300 | 0.0381 | | | | | |
| NH14 | | 03.01.01 | 68.01.02 | 35.01.01 | 44.02.01 | 46 | 67 | 0.0120 | 0.2200 | 0.2320 | 192 | 59 | 0.0084 | 0.1200 | 0.1284 | 384 | 92 | 0.0100 | 0.0029 | 0.0129 |
| NH17 | D614G (20C) | 02.01.01 | 24.02.01 | 18.01.01 | 44.02.01 | 24 | 937 | 0.0310 | 0.0320 | 0.0630 | 178 | 109 | 0.0160 | 0.0150 | 0.0310 | | | | | |
| NH20 | | 02.01.01 | | 07.02.01 | 50.01.01 | 49 | 335 | 0.0130 | 0.0180 | 0.0310 | 186 | 139 | 0.0190 | 0.0240 | 0.0430 | | | | | |
| NH21 | | 02.01.01 | 03.01.01 | 07.02.01 | 40.01.02 | 76 | 80 | 0.4500 | 0.2400 | 0.6900 | 187 | 53 | 0.1800 | 0.0430 | 0.2230 | | | | | |
| NH22 | | 24.02.01 | 03.01.01 | 27.05.02 | 35.03.01 | 101 | 88 | 0.0230 | 0.0900 | 0.1130 | 192 | 78 | 0.0092 | 0.0570 | 0.0662 | | | | | |
| NH23 | | 01.01.01 | 03.01.01 | 07.02.01 | 08.01.01 | 82 | 169 | 0.0420 | 0.1800 | 0.2220 | 201 | 203 | 0.0320 | 0.0620 | 0.0940 | | | | | |
| NH25 | D614G (20C) | 02.01.01 | | 07.02.01 | 50.01.01 | 45 | 423 | 0.0410 | 0.0340 | 0.0750 | 162 | 170 | 0.0490 | 0.0240 | 0.0730 | | | | | |
| NH26 | | 24.02.01 | 29.02.01 | 15.01.01 | 39.06.02 | 71 | 125 | 0.0910 | 0.8000 | 0.8910 | 188 | 130 | 0.0140 | 0.2800 | 0.2940 | | | | | |
| NH27 | | 02.01.01 | 03.01.01 | 15.01.01 | | 85 | 116 | 0.0072 | 0.0072 | 0.0143 | 190 | 79 | 0.0053 | 0.0098 | 0.0151 | 380 | 1098 | 0.0340 | 0.0160 | 0.0500 |
| NH28 | | 02.01.01 | 01.01.01 | 27.05.02 | 08.01.01 | 105 | 55 | 0.1300 | 0.0160 | 0.1460 | 195 | 73 | 0.0370 | 0.0052 | 0.0422 | | | | | |
| NH29 | D614G (20C) | 24.02.01 | 01.01.01 | 37.01.01 | 40.01.02 | 49 | 71 | 0.0028 | 0.0160 | 0.0188 | 224 | 23 | 0.0058 | 0.0160 | 0.0218 | | | | | |
| NH31 | D614G (20C) | 02.01.01 | 11.01.01 | 15.01.01 | 35.01.01 | 90 | 65 | 0.0220 | 0.2200 | 0.2420 | 192 | 27 | 0.0053 | 0.2000 | 0.2053 | 353 | 154 | 0.0370 | 0.0041 | 0.0411 |
| NH32 | | 26.01.01 | 11.01.01 | 35.01.01 | 38.01.01 | 28 | 112 | 0.3100 | 0.4200 | 0.7300 | 182 | 70 | 0.3500 | 0.1500 | 0.5000 | | | | | |
| NH33 | | 24.02.01 | 30.01.01 | 07.02.01 | 13.02.01 | 20 | 264 | 0.0079 | 0.0330 | 0.0409 | 190 | 120 | 0.0110 | 0.0150 | 0.0260 | | | | | |
| NH34 | | 02.01.01 | 03.01.01 | 07.02.01 | 15.01.01 | 25 | 310 | 0.0120 | 0.0470 | 0.0590 | 180 | 150 | 0.0026 | 0.0160 | 0.0186 | 383 | 81 | 0.0150 | 0.1200 | 0.1350 |
| NH38 | | 02.01.01 | 01.01.01 | 51.01.01 | 08.01.01 | 29 | 406 | 0.7700 | 0.1500 | 0.9200 | 195 | 235 | 0.5300 | 0.0260 | 0.5560 | 372 | 213 | 0.0070 | 0.0630 | 0.0700 |
| NH39 | D614G (20C) | 02.01.01 | 26.01.01 | 40.01.01 | 35.01.01 | 77 | 253 | 0.1100 | 0.4200 | 0.5300 | 174 | 125 | 0.0110 | 0.1500 | 0.1610 | | | | | |
| NH40 | | 02.01.01 | | 44.02.01 | 15.01.01 | 86 | 272 | 0.0480 | 0.0730 | 0.1210 | 182 | 165 | 0.0190 | 0.0380 | 0.0570 | 371 | 260 | 0.0100 | 0.0160 | 0.0260 |
| NH43 | | 02.01.01 | 11.01.01 | 44.02.01 | 07.01.01 | 85 | 331 | 0.0091 | 0.0180 | 0.0271 | 190 | 250 | 0.0130 | 0.0110 | 0.0240 | | | | | |
| NH45 | D614G (20C) | 02.01.01 | 03.01.01 | 20.05.01 | 51.01.01 | 86 | 120 | 0.0220 | 0.0150 | 0.0370 | 175 | 189 | 0.0130 | 0.0120 | 0.0250 | | | | | |
| NH47 | | 25.03.01 | 02.01.01 | 41.01.01 | 18.01.02 | 42 | 158 | 0.0150 | 0.2000 | 0.2150 | 182 | 97 | 0.0120 | 0.1200 | 0.1320 | | | | | |
| NH48 | | 02.01.01 | 32.01.01 | 40.01.02 | 44.02.01 | 141 | 118 | 0.0260 | 0.0590 | 0.0850 | 183 | 150 | 0.0240 | 0.0440 | 0.0680 | | | | | |
| NH50 | | 03.01.01 | 30.01.01 | 49.01.01 | 55.01.01 | 77 | 148 | 0.0077 | 0.0052 | 0.0129 | 186 | 129 | 0.0085 | 0.0070 | 0.0155 | | | | | |
| NH51 | | 02.01.01 | | 18.01.01 | 40.01.02 | 138 | 1670 | 0.2400 | 0.0400 | 0.2800 | 189 | 1313 | 0.0610 | 0.0190 | 0.0800 | | | | | |
| NH52 | | 11.01.01 | 01.01.01 | 15.01.01 | 08.01.01 | 68 | 132 | 0.0160 | 0.0930 | 0.1090 | 182 | 106 | 0.0350 | 0.0560 | 0.0910 | 357 | 65 | 0.0053 | 0.0420 | 0.0473 |

**Supplementary Table 1.** Summary of the 28 study participants. Subjects have their HLA Typing for A and B described, with green marking for those that show match with the Dextramer reagents described Supplementary Table 2. Samples taken at Baseline, 6M and 12M are characterized by Days post onset of disease, antibody $ID_{50}$, $CD8^+$ T-cells frequency (%) for spike, Non-spike and total epitopes for each sample. Samples for 12M marked in grey were omitted due to vaccination after the 6M sample was taken. The virus infecting variant detected for 6 participants with viral genomes sequenced are shown.

| Dextramer no. | Peptide sequence | HLA allele | Target virus | Target protein | Fluorophore |
|---|---|---|---|---|---|
| 1 | LTDEMIAQY | A*01:01 | SARS-CoV-2 | Spike | PE |
| 2 | WTAGAAAYY | A*01:01 | SARS-CoV-2 | Spike | PE |
| 3 | CTDDNALAYY | A*01:01 | SARS-CoV-2 | ORF1ab | APC |
| 4 | TTDPSFLGRY | A*01:01 | SARS-CoV-2 | ORF1ab | APC |
| 5 | FTSDYYQLY | A*01:01 | SARS-CoV-2 | ORF3a | APC |
| 6 | VTEHDTLLY | A*01:01 | CMV | DNA polymerase | PE |
| 7 | STEGGGLAY | A*01:01 | Non-specific | N/A | APC |
| 8 | YLQPRTFLL | A*02:01 | SARS-CoV-2 | Spike | PE |
| 9 | NLNESLIDL | A*02:01 | SARS-CoV-2 | Spike | PE |
| 10 | FIAGLIAIV | A*02:01 | SARS-CoV-2 | Spike | PE |
| 11 | ALWEIQQVV | A*02:01 | SARS-CoV-2 | ORF1ab | APC |
| 12 | LLLDRLNQL | A*02:01 | SARS-CoV-2 | Nucelocapsid | APC |
| 13 | LLYDANYFL | A*02:01 | SARS-CoV-2 | ORF3a | APC |
| 14 | GLCTLVAML | A*02:01 | EBV | mRNA export factor (ICP27) | PE |
| 15 | GILGFVFTL | A*02:01 | Influenza | Matrix protein 1 (MP1) | PE |
| 16 | ALIAPVHAV | A*02:01 | Non-specific | N/A | APC |
| 17 | KCYGVSPTK | A*03:01 | SARS-CoV-2 | Spike | PE |
| 18 | GVYFASTEK | A*03:01 | SARS-CoV-2 | Spike | PE |
| 19 | KTFPPTEPK | A*03:01 | SARS-CoV-2 | Nucelocapsid | APC |
| 20 | KTIQPRVEK | A*03:01 | SARS-CoV-2 | ORF1ab | APC |
| 21 | VVYRGTTTYK | A*03:01 | SARS-CoV-2 | ORF1ab | APC |
| 22 | KLGGALQAK | A*03:01 | CMV | Immediate early protein (IE1) | PE |
| 23 | GLFGAGAFK | A*03:01 | Non-specific | N/A | APC |
| 24 | KCYGVSPTK | A*11:01 | SARS-CoV-2 | Spike | PE |
| 25 | RLFRKSNLK | A*11:01 | SARS-CoV-2 | Spike | PE |
| 26 | GVYFASTEK | A*11:01 | SARS-CoV-2 | Spike | PE |
| 27 | ASMPTTIAK | A*11:01 | SARS-CoV-2 | ORF1ab | APC |
| 28 | ATEGALNTPK | A*11:01 | SARS-CoV-2 | Nucelocapsid | APC |
| 29 | KTFPPTEPK | A*11:01 | SARS-CoV-2 | Nucelocapsid | APC |
| 30 | ATVQGQNLK | A*11:01 | CMV | pp65 | PE |
| 31 | QYIKWPWYI | A*24:02 | SARS-CoV-2 | Spike | PE |
| 32 | NYNYLYRLF | A*24:02 | SARS-CoV-2 | Spike | PE |
| 33 | VYFLQSINF | A*24:02 | SARS-CoV-2 | Nucelocapsid | APC |
| 34 | VYIGDPAQL | A*24:02 | SARS-CoV-2 | ORF1ab | APC |
| 35 | QYDPVAALF | A*24:02 | CMV | Phosphoprotein | PE |
| 36 | SPRRARSVA | B*07:02 | SARS-CoV-2 | Spike | PE |
| 37 | IPRRNVATL | B*07:02 | SARS-CoV-2 | ORF1ab | APC |
| 38 | KPRQKRTAT | B*07:02 | SARS-CoV-2 | Nucelocapsid | APC |
| 39 | SPRQYFYYL | B*07:02 | SARS-CoV-2 | Nucelocapsid | APC |
| 40 | TPRVTGGGAM | B*07:02 | CMV | Phosphoprotein | PE |
| 41 | ELRRKMMYM | B*08:01 | CMV | Immediate early protein (IE1) | PE |
| 42 | QPTESIVRF | B*35:01 | SARS-CoV-2 | Spike | PE |
| 43 | LPFNDGVYF | B*35:01 | SARS-CoV-2 | Spike | PE |
| 44 | IPFAMQMAY | B*35:01 | SARS-CoV-2 | Spike | PE |
| 45 | IPSINVHHY | B*35:01 | CMV | Phosphoprotein | PE |

**Supplementary Table 2.** Overview of MHC class I Dextramer reagents used for flowcytometry in this study**.** The table depicts the Dextramer composition with regards to peptide sequence, HLA allele presentation, target virus, target viral protein and fluorophore for flow identification.

| Subject ID | HLA type | | | | Dextramer pool | |
|---|---|---|---|---|---|---|
| | A | | B | | Tube 1 | Tube 2 |
| NH07 | 01:01:01 | 02:01:01 | 08:01:01 | 18:01:01 | 1-5,8-13 | 6,7,14-16, 41 |
| NH09 | 01:01:01 | 32:01:01 | 08:01:01 | 40:02:01 | 1-5 | 6,7,41 |
| NH13 | 02:01:01 | 11:01:01 | 35:01:01 | 52:01:01 | 8-13, 24-29, 42-44 | 14-16, 30, 45 |
| NH14 | 03:01:01 | 68:01:02 | 35:01:01 | 44:02:01 | 17-21, 42-44 | 22, 23, 45 |
| NH17 | 02:01:01 | 24:02:01 | 18:01:01 | 44:02:01 | 8-13, 31-34 | 14-16, 35 |
| NH20 | 02:01:01 | | 07:02:01 | 50:01:01 | 8-13, 36-39 | 14-16, 40 |
| NH21 | 02:01:01 | 03:01:01 | 07:02:01 | 40:01:02 | 8-13, 17-21, 36-39 | 14-16, 22,23,40 |
| NH22 | 24:02:01 | 03:01:01 | 27:05:02 | 35:03:01 | 31-34, 17-21 | 22, 23, 35 |
| NH23 | 01:01:01 | 03:01:01 | 07:02:01 | 08:01:01 | 1-5, 17-21, 36-39 | 6, 7, 22 ,23, 40, 41 |
| NH25 | 02:01:01 | | 07:02:01 | 50:01:01 | 8-13, 36-39 | 14-16, 40 |
| NH26 | 24:02:01 | 29:02:01 | 15:01:01 | 39:06:02 | 31-34 | 35 |
| NH27 | 02:01:01 | 03:01:01 | 15:01:01 | | 8-13, 17-21 | 14-16, 22, 23 |
| NH28 | 02:01:01 | 01:01:01 | 27:05:02 | 08:01:01 | 1-5, 8-13 | 6, 7, 14-16, 41 |
| NH29 | 24:02:01 | 01:01:01 | 37:01:01 | 40:01:02 | 1-5, 31-34 | 6, 7, 35 |
| NH31 | 02:01:01 | 11:01:01 | 15:01:01 | 35:01:01 | 8-13, 24-29, 42-44 | 14-16, 30, 45 |
| NH32 | 26:01:01 | 11:01:01 | 35:01:01 | 38:01:01 | 24-29, 42-44 | 30, 45 |
| NH33 | 24:02:01 | 30:01:01 | 07:02:01 | 13:02:01 | 31-34, 36-39 | 35, 45 |
| NH34 | 02:01:01 | 03:01:01 | 07:02:01 | 15:01:01 | 8-13, 17-21, 36-39 | 14-16, 22, 23, 40 |
| NH38 | 02:01:01 | 01:01:01 | 51:01:01 | 08:01:01 | 1-5, 8-13 | 6, 7, 14-16, 41 |
| NH39 | 02:01:01 | 26:01:01 | 40:01:01 | 35:01:01 | 8-13, 42-44 | 14-16, 45 |
| NH40 | 02:01:01 | | 44:02:01 | 15:01:01 | 8-13 | 14-16 |
| NH43 | 02.01.01 | 11.01.01 | 44:02:01 | 07:02:01 | 8-13, 24-29, 36-39 | 14-16, 30, 40 |
| NH45 | 02.01.01 | 03.01.01 | 20:05:01 | 51:01:01 | 8-13, 17-21 | 14-16, 22, 23 |
| NH47 | 25:03:01 | 02:01:01 | 41:01:01 | 18:01:02 | 8-13 | 14-16 |
| NH48 | 02:01:01 | 32:01:01 | 40:01:02 | 44:02:01 | 8-13 | 14-16 |
| NH50 | 03:01:01 | 30:01:01 | 49:01:01 | 55:01:01 | 17-21 | 22, 23 |
| NH51 | 02:01:01 | | 18:01:01 | 40:01:02 | 8-13 | 14-16 |
| NH52 | 11:01:01 | 01:01:01 | 15:01:01 | 08:01:01 | 1-5, 24-29 | 6, 7, 30, 41 |

**Supplementary Table 3.** Specific pools of Dextramer reagents used for each participant. Subjects have their HLA Typing for A and B described, with green marking for those that show match with the Dextramer reagents described Supplementary Table 2. Two pools of dextramers for each sample were generated as described column four and five referring to Dextramer numbers in Supplementary Table 2.

| Dextramer no. | Peptide sequence | HLA allele | Target virus | Target protein | Fluorophore |
|---|---|---|---|---|---|
| 10x-MHC-I-1 | LTDEMIAQY | A*01:01 | SARS-CoV-2 | Spike | PE |
| 10x-MHC-I-2 | WTAGAAAYY | A*01:01 | SARS-CoV-2 | Spike | PE |
| 10x-MHC-I-3 | CTDDNALAYY | A*01:01 | SARS-CoV-2 | ORF1ab | PE |
| 10x-MHC-I-4 | TTDPSFLGRY | A*01:01 | SARS-CoV-2 | ORF1ab | PE |
| 10x-MHC-I-5 | FTSDYYQLY | A*01:01 | SARS-CoV-2 | ORF3a | PE |
| 10x-MHC-I-6 | VTEHDTLLY | A*01:01 | CMV | DNA polymerase | PE |
| 10x-MHC-I-7 | STEGGGLAY | A*01:01 | Non-specific | N/A | PE |
| 10x-MHC-I-8 | YLQPRTFLL | A*02:01 | SARS-CoV-2 | Spike | PE |
| 10x-MHC-I-9 | NLNESLIDL | A*02:01 | SARS-CoV-2 | Spike | PE |
| 10x-MHC-I-10 | FIAGLIAIV | A*02:01 | SARS-CoV-2 | Spike | PE |
| 10x-MHC-I-11 | ALWEIQQVV | A*02:01 | SARS-CoV-2 | ORF1ab | PE |
| 10x-MHC-I-12 | LLLDRLNQL | A*02:01 | SARS-CoV-2 | Nucelocapsid | PE |
| 10x-MHC-I-13 | LLYDANYFL | A*02:01 | SARS-CoV-2 | ORF3a | PE |
| 10x-MHC-I-14 | GLCTLVAML | A*02:01 | EBV | mRNA export factor (ICP27) | PE |
| 10x-MHC-I-15 | GILGFVFTL | A*02:01 | Influenza | Matrix protein 1 (MP1) | PE |
| 10x-MHC-I-16 | ALIAPVHAV | A*02:01 | Non-specific | N/A | PE |
| 10x-MHC-I-17 | KCYGVSPTK | A*03:01 | SARS-CoV-2 | Spike | PE |
| 10x-MHC-I-18 | GVYFASTEK | A*03:01 | SARS-CoV-2 | Spike | PE |
| 10x-MHC-I-19 | KTFPPTEPK | A*03:01 | SARS-CoV-2 | Nucelocapsid | PE |
| 10x-MHC-I-20 | KTIQPRVEK | A*03:01 | SARS-CoV-2 | ORF1ab | PE |
| 10x-MHC-I-21 | VVYRGTTTYK | A*03:01 | SARS-CoV-2 | ORF1ab | PE |
| 10x-MHC-I-22 | KLGGALQAK | A*03:01 | CMV | Immediate early protein (IE1) | PE |
| 10x-MHC-I-23 | GLFGAGAFK | A*03:01 | Non-specific | N/A | PE |
| 10x-MHC-I-24 | KCYGVSPTK | A*11:01 | SARS-CoV-2 | Spike | PE |
| 10x-MHC-I-25 | RLFRKSNLK | A*11:01 | SARS-CoV-2 | Spike | PE |
| 10x-MHC-I-26 | GVYFASTEK | A*11:01 | SARS-CoV-2 | Spike | PE |
| 10x-MHC-I-27 | ASMPTTIAK | A*11:01 | SARS-CoV-2 | ORF1ab | PE |
| 10x-MHC-I-28 | ATEGALNTPK | A*11:01 | SARS-CoV-2 | Nucelocapsid | PE |
| 10x-MHC-I-29 | KTFPPTEPK | A*11:01 | SARS-CoV-2 | Nucelocapsid | PE |
| 10x-MHC-I-30 | ATVQGQNLK | A*11:01 | CMV | pp65 | PE |

**Supplementary Table 4.** Overview of MHC class I Dextramer reagents for 10x used in this study. The table describes the Dextramer composition with regards to HLA allele presentation, peptide sequence, Target Virus, Target viral Protein and Fluorophore for flow identification.

| Dextramer no. | HLA allele | Peptide sequence | Target virus | Target protein | Fluorophore |
|---|---|---|---|---|---|
| 10x-MHC-II-1 | DRB1*0401 | QLIRAAEIRASANLAATK | SARS-CoV-2 | Spike | PE |
| 10x-MHC-II-2 | DRB1*0401 | MAYRFNGIGVTQNVLY | SARS-CoV-2 | Spike | PE |
| 10x-MHC-II-3 | DRB1*0401 | QALNTLVKQLSSNFGAI | SARS-CoV-2 | Spike | PE |
| 10x-MHC-II-4 | DRB1*0401 | KSFTVEKGIYQTSNFRVQ | SARS-CoV-2 | Spike | PE |
| 10x-MHC-II-5 | DRB1*0401 | HWFVTQRNFYEPQII | SARS-CoV-2 | Spike | PE |
| 10x-MHC-II-6 | DRB1*0401 | QSIIAYTMSLGAENSVAY | SARS-CoV-2 | Spike | PE |
| 10x-MHC-II-7 | DRB1*0401 | DSLSSTASALGKLQDVV | SARS-CoV-2 | Spike | PE |
| 10x-MHC-II-8 | DRB1*0401 | LEASFNYLKSPNFSK | SARS-CoV-2 | Orf1ab | PE |
| 10x-MHC-II-9 | DRB1*0401 | FYVYSRVKNLNSSRV | SARS-CoV-2 | E | PE |
| 10x-MHC-II-10 | DRB1*0401 | ESPFVMMSAPPAQYE | SARS-CoV-2 | Orf1ab | PE |
| 10x-MHC-II-11 | DRB1*0401 | EEIAIILASFSASTS | SARS-CoV-2 | Orf1ab | PE |
| 10x-MHC-II-12 | DRB1*0401 | EAFEKMVSLLSVLLS | SARS-CoV-2 | Orf1ab | PE |
| 10x-MHC-II-13 | DRB1*0401 | PVSKMRMATPLLMQA | Non-specific | N/A | PE |
| 10x-MHC-II-14 | DRB1*0701 | QLIRAAEIRASANLAATK | SARS-CoV-2 | Spike | PE |
| 10x-MHC-II-15 | DRB1*0701 | GAALQIPFAMQMAYRF | SARS-CoV-2 | Spike | PE |
| 10x-MHC-II-16 | DRB1*0701 | MAYRFNGIGVTQNVLY | SARS-CoV-2 | Spike | PE |
| 10x-MHC-II-17 | DRB1*0701 | SASFSTFKCYGVSPTKL | SARS-CoV-2 | Spike | PE |
| 10x-MHC-II-18 | DRB1*0701 | KSFTVEKGIYQTSNFRVQ | SARS-CoV-2 | Spike | PE |
| 10x-MHC-II-19 | DRB1*0701 | VKQIYKTPPIKDFGGFNF | SARS-CoV-2 | Spike | PE |
| 10x-MHC-II-20 | DRB1*0701 | HWFVTQRNFYEPQII | SARS-CoV-2 | Spike | PE |
| 10x-MHC-II-21 | DRB1*0701 | QSIIAYTMSLGAENSVAY | SARS-CoV-2 | Spike | PE |
| 10x-MHC-II-22 | DRB1*0701 | LEASFNYLKSPNFSK | SARS-CoV-2 | Orf1ab | PE |
| 10x-MHC-II-23 | DRB1*0701 | FYVYSRVKNLNSSRV | SARS-CoV-2 | E | PE |
| 10x-MHC-II-24 | DRB1*0701 | EEIAIILASFSASTS | SARS-CoV-2 | Orf1ab | PE |
| 10x-MHC-II-25 | DRB1*0701 | EAFEKMVSLLSVLLS | SARS-CoV-2 | Orf1ab | PE |
| 10x-MHC-II-26 | DRB1*0701 | PVSKMRMATPLLMQA | Non-specific | N/A | PE |

**Supplementary Table 5.** Overview of MHC class II Dextramer reagents for 10x used in this study. The table describes the Dextramer composition with regards to HLA allele presentation, peptide sequence, Target Virus, Target viral Protein and Fluorophore for flow identification.
